# Adherence to and Engagement With an mHealth Physical Activity Intervention After Mild Stroke or Transient Ischemic Attack: Secondary Analysis of a Feasibility Randomized Controlled Trial

**DOI:** 10.2196/75662

**Published:** 2026-03-17

**Authors:** Hanna Lagerlund, Lucian Bezuidenhout, Sophia Humphries, Lisa Holmlund, Lydia Kwak, Charlotte K Häger, David Moulaee Conradsson

**Affiliations:** 1 Division of Physiotherapy, Department of Neurobiology, Care Sciences and Society, Karolinska Institutet, Alfred Nobels Allé 23, 23100, Stockholm, SE-141 52, Sweden, 46 852486614; 2 Division of Occupational Therapy, Department of Neurobiology, Care Sciences and Society, Karolinska Institutet, Stockholm, Sweden; 3 Unit of Intervention and Implementation Research for Worker Health, Institute of Environmental Medicine, Karolinska Institutet, Stockholm, Sweden; 4 Department of Community Medicine and Rehabilitation, Umeå University, Umeå, Sweden; 5 Department of Diagnostics and Intervention, Umeå University, Umeå, Sweden; 6 Medical Unit Allied Health Professionals, Women’s Health and Allied Health Professionals Theme, Karolinska University Hospital, Stockholm, Sweden

**Keywords:** digital interventions, digital health, mobile health, mobile applications, secondary prevention, exercise, engagement, adherence

## Abstract

**Background:**

Regular physical activity is a crucial and an important modifiable lifestyle factor reducing the risk of recurrent incidents after stroke or transient ischemic attack (TIA). Mobile health (mHealth) has emerged as a promising approach for providing long-term support for physical activity. However, little is known about how individuals poststroke or TIA adhere to and engage with mHealth interventions.

**Objective:**

This study aimed to (1) describe adherence to supervised sessions in an mHealth intervention targeting physical activity, (2) describe engagement with self-managed mHealth support for physical activity during and after the intervention, (3) compare characteristics of participants with high and low adherence and app engagement, and (4) examine whether high adherence and app engagement were associated with maintained physical activity after having completed the intervention and at a 12-month follow-up.

**Methods:**

In this study, a secondary analysis of data from the experimental arm of a feasibility randomized controlled trial was conducted. The experimental group received a 6-month mHealth version of the i-REBOUND intervention, which included supervised mHealth support for physical activity and behavior change, followed by a 6-month postintervention period with access to self-managed mHealth support. The control group received mHealth consultations via video conferencing. Adherence measures included attendance at supervised exercise and counseling sessions, while app engagement was measured by weekly interactions with self-managed mHealth support during and after the intervention. Participants’ level of physical activity (steps per day) was measured using accelerometers at baseline, and at 6- and 12-month postbaseline. Logistic regression analysis examined the associations between high adherence and app engagement during the intervention and postintervention period and maintained physical activity (ie, >7000 steps/day) across the 12-month study period.

**Results:**

Of the 57 participants enrolled, 51 (89%) completed the intervention; the average age was 71 years, 34/51 (67%) were female, and 47/51 (92%) had mild stroke symptoms. Adherence to supervised mHealth support was high (supervised exercise sessions: 79%, counseling sessions: 98%), while engagement with self-managed mHealth support was high during the intervention (83%) but declined postintervention (38%). A larger proportion of females (24/31, 77%) demonstrated high adherence to the intervention compared to males (7/31, 23%, *χ*²_1_=4.1; *P*=.04). High adherence (≥80%) during the intervention was associated with maintained physical activity between baseline and the 6-month follow-up (OR 12.07, 95% CI 2‐72.76; *P*=.01), while high app engagement (≥80%) during postintervention was associated with maintained physical activity between the 6- and 12-month follow-up (OR 5.10, 95% CI 1.02‐25.52; *P*=.05).

**Conclusions:**

Supervised mHealth support was well received with high adherence, while modules for self-management of physical activity faced challenges in engaging the participants. Future studies could benefit from qualitative and cocreative approaches to better understand and refine self-managed mHealth support for individuals poststroke or TIA.

## Introduction

Stroke affects 15 million people annually and is the third leading cause of death and disability worldwide [1]. A previous stroke or transient ischemic attack (TIA) increases the risk of a recurrent stroke, often with more severe health consequences [2]. Physical activity is an important modifiable lifestyle factor that contributes to a risk reduction of recurrent stroke and long-term disability for up to five years after a mild stroke or TIA [3]. Despite the crucial role of physical activity in secondary prevention [2], access to long-term support for physical activity after a stroke or TIA remains limited [4,5]. Many individuals poststroke fail to meet recommended physical activity levels, spend extended periods sedentary [6], and often require therapist-led support to maintain long-term engagement in physical activity.

Interventions combining onsite supervised physical exercise with support for physical activity through behavior change techniques appear to be particularly effective in improving cardiovascular health (eg, systolic blood pressure) [7]. However, barriers such as lack of access to professional support, long travel times, and inadequate public transportation limit people poststroke or TIA from attending on-site physical activity services [8,9]. Mobile health (mHealth), that is, health care delivered via mobile devices, is a promising intervention modality for promoting physical activity poststroke [10], offering a flexible solution with the potential to support long-term physical activity. Yet, individuals poststroke may face challenges in using mHealth technology due to limited digital literacy and stroke-related impairments such as fatigue, lack of motor skills, or cognitive difficulties, which can hinder their ability to effectively interact with mobile devices [11]. Despite these challenges, individuals poststroke are generally satisfied with digital services and show a willingness to engage with technology-based support, indicating that mHealth may be a feasible and acceptable option for this population [12]. In the context of secondary stroke prevention, mHealth provides an underutilized opportunity to enhance access to physical activity support and reduce the risk of secondary complications after stroke or TIA [10,13].

The health outcomes of digital interventions, such as mHealth, are influenced by the individuals’ adherence to (ie, the extent to which the participant followed the intervention as designed) and engagement with the intervention [14-16]. Engagement is a multifaceted concept involving both *what* users are engaged with (ie, the targeted health behavior and the digital intervention) and *how* they engage with the digital intervention [17,18]. In terms of *how*, Perski et al [19] conceptualize engagement as having both behavioral and experiential dimensions. The behavioral dimension refers to the extent of active interaction with the intervention, while the experiential dimension encompasses subjective experiences such as feelings and interest [19]. A recent scoping review suggested higher adherence to mHealth interventions among males, older individuals, and those with secondary education [15]. Limited research has explored adherence to and engagement with mHealth interventions in individuals poststroke or TIA [10], and the factors influencing these behaviors remain largely unknown. A better understanding of adherence and app engagement can inform future development of mHealth interventions to promote and maintain physical activity poststroke or TIA.

This study builds on “i-REBOUND—let’s get moving,” a telehealth intervention developed in Australia to support home-based exercise and the promotion and maintenance of physical activity in people poststroke or TIA [20]. To improve reach, i-REBOUND was developed into a fully digital mHealth intervention [21], which provides 6 months of supervised support for physical activity and behavior change, followed by a 6-month postintervention period with access to self-managed mHealth support. Effective mHealth interventions depend on individuals’ adherence and app engagement to ensure sufficient exposure to the intervention and, ultimately, to achieving the health outcomes. Thus, optimizing and tailoring support for future mHealth interventions requires a deeper understanding of how individuals adhere to and engage with these technologies. While a previous feasibility trial demonstrated that the i-REBOUND intervention was safe, acceptable, and accessible for people poststroke or TIA across Sweden [22], it did not assess participant adherence or engagement over time, nor the association with physical activity outcomes. Therefore, the present secondary analysis examines patterns of adherence to and engagement with the mHealth intervention and the association with maintained physical activity after the intervention. The specific aims were to (1) describe adherence to supervised exercise and individual counseling sessions during the intervention, (2) describe engagement with the self-managed mHealth support for physical activity during intervention and postintervention, (3) compare the characteristics of participants with high and low levels of adherence and engagement in the mHealth intervention, and (4) examine whether high adherence and app engagement were associated with maintained physical activity between baseline and the 6-month follow-up, and between the 6- and 12-month follow-up.

## Methods

### Study Design

The present study was a preplanned secondary analysis of data from the experimental arm of a feasibility randomized controlled trial (ClinicalTrials.gov: NCT0511195). The trial included individuals poststroke or TIA randomized into an experimental group receiving the mHealth version of the i-REBOUND intervention or a control group receiving mHealth consultations via videoconferencing [21,22]. The analysis focused on participants’ adherence to and engagement with the mHealth intervention and was therefore limited to the experimental arm. The data were collected between September 2021 and December 2023. This study was reported in accordance with the CONSORT-EHEALTH (Consolidated Standards of Reporting Trials of Electronic and Mobile Health Applications and Online Telehealth) checklist (Checklist 1) [23].

### Ethical Considerations

The study was approved by the Swedish Ethical Review Authority (dnr 2020‐05062 and 2021‐03622). Participation in the study was voluntary, and participants could withdraw at any time without consequence. No compensation was provided. Individuals who expressed interest received both verbal and written information before providing written informed consent. This consent also included approval for the secondary analysis conducted in the present study. A detailed description of the inclusion procedure and ethical considerations is available in the study protocol [21]. All data were anonymized to ensure participant confidentiality.

### Participants

Participants were recruited through advertisements at collaborating clinics, social media, patient organizations, and the webpage of Karolinska Institutet. Inclusion criteria were (1) clinical diagnosis of stroke or TIA between 3 months and 10 years prior to study enrollment, confirmed by the participants’ physician, (2) living at home, (3) being able to walk a short distance indoors with or without a walking device, (4) being able to use a smartphone including e-signature identification with or without the help of a relative or carer, and (5) having access to a stable internet connection. Exclusion criteria were (1) already meeting the recommended physical activity levels of at least 150 minutes per week of moderate physical activity or at least 75 minutes per week of vigorous-intensity physical activity, (2) severe health conditions compromising engagement in the intervention, or (3) enrolled in a concomitant clinical trial or participating in rehabilitation (eg, aerobic exercises) at the time point of recruitment.

### Intervention

#### The mHealth Version of the i-REBOUND Intervention

**Figure 1.**
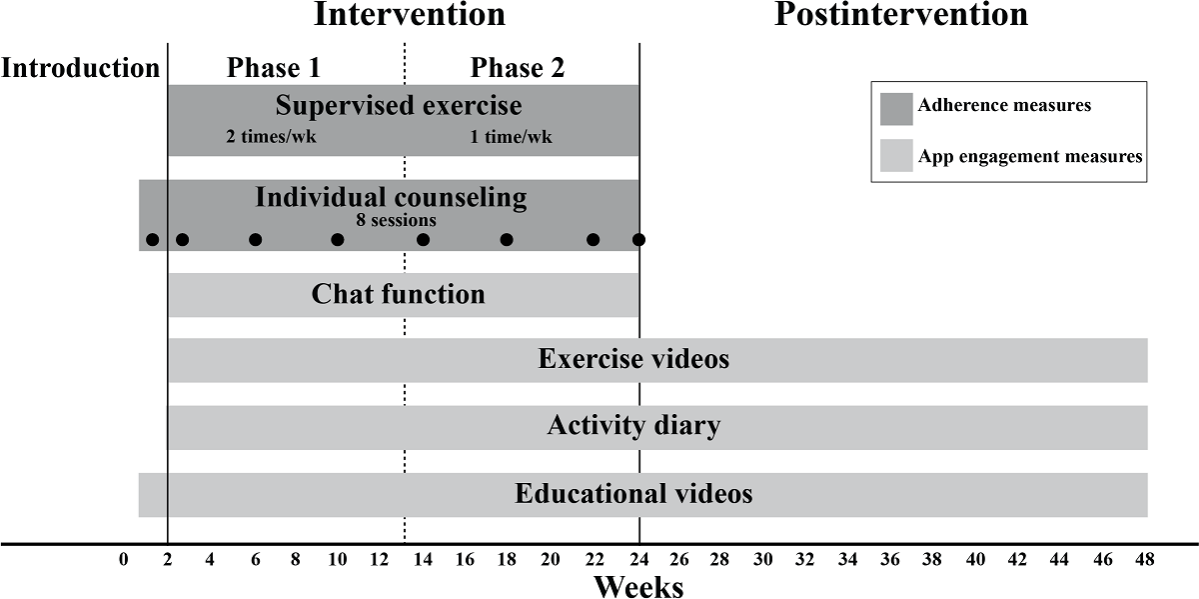
Illustration of the mobile health intervention and postintervention across the 48-week study period.

**Figure 2.**
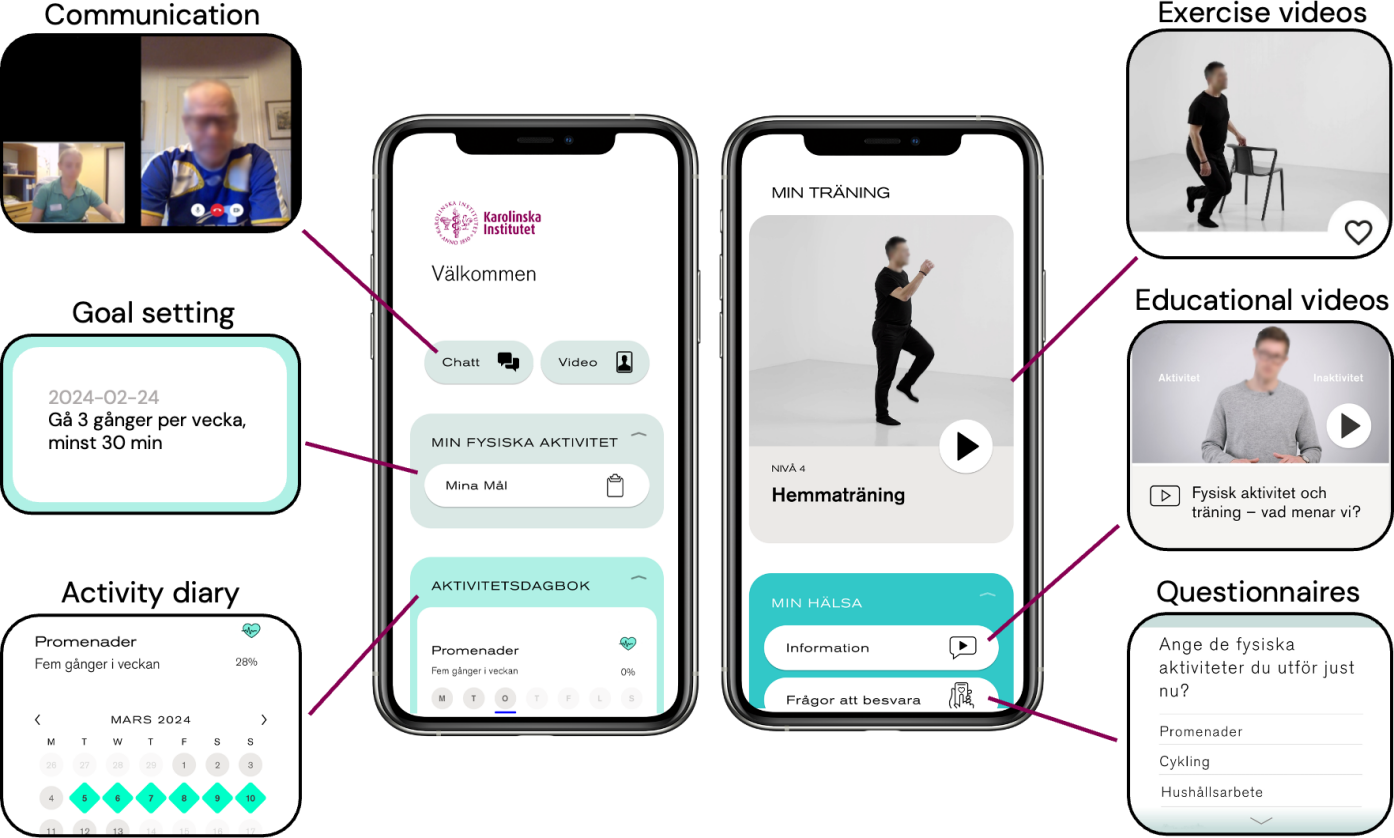
Overview of the mobile health intervention components and the Stroke Treatment Through Active and Accessible Rehabilitation (STAAR) app’s interface.

The mHealth intervention provided support for physical activity and behavior change to individuals poststroke or TIA across 48 weeks, divided into an intervention and postintervention period (Figure 1). Full intervention details are outlined in the study protocol [21]. The intervention was delivered via the STAAR (Stroke Treatment Through Active and Accessible Rehabilitation) app, which was managed by the medical technology company Empowered Health and available on iOS and Android devices. Participants accessed all intervention components through the app, including an overview of their individual goals, educational videos, an activity diary for self-monitoring, and prerecorded exercise videos (Figure 2). Supervised sessions were delivered via video calls within the app, and written communication occurred through a chat function. Two physical therapists with at least 5 years of experience in stroke rehabilitation delivered the intervention through a web-based digital clinic connected to the app. Baseline assessments were conducted using digital questionnaires administered via the app.

#### Supervised mHealth Support

During the first week, the mHealth intervention was introduced in 2 sessions with the physical therapist, including a medical history review, discussions about resources for physical activity and home exercises, and a practice exercise session. Phase 1 of the intervention (wk 2‐12) offered 2 supervised exercise sessions per week, and phase 2 (wk 13‐24) offered 1 session. The supervised 30-minute exercise sessions aimed to reach physical activity at moderate intensity (ie, Borg Rating of Perceived Exercise Exertion Scale >12 [24]) and were delivered individually or in groups based on participants’ physical capacity.

Support for behavior change consisted of individual monthly counseling sessions with the physical therapist. These sessions included discussions on goals, self-management strategies, and barriers and facilitators to physical activity. In addition to the supervised exercise sessions, participants were encouraged to engage in any physical activity of their choice. The final counseling session during the last week of the intervention period focused on developing an individualized self-management plan and setting goals for the postintervention period, supporting maintenance of exercise routines and engagement in physical activity.

#### Self-Managed mHealth Support

Educational videos on physical activity and exercise recommendations, individually prescribed exercise videos, and an activity diary were available throughout the intervention and postintervention period as behavioral change techniques to support the promotion of physical activity in analogy with the behavior change technique taxonomy (v1) by Michie et al [25] (see Multimedia Appendix 1). Participants were encouraged to engage with the self-managed mHealth support across the intervention and postintervention periods, although no specific instructions for intended usage were provided. Individual exercises, with prerecorded instructional videos for strength and/or aerobic training, were prescribed by the physical therapist based on the needs and preferences of each participant. The digital activity diary encouraged self-management by enabling participants to self-monitor their physical activity. Participants selected activities to monitor, such as outdoor walking, and designated days to perform them, marking each activity as “performed” or “not performed” in the diary. Participants had the option to receive mobile phone reminders for the selected activities through the activity diary. During postintervention, the study participants had access to self-managed mHealth support, but no physical therapist contact was offered.

### Measures

#### Baseline Measures

Baseline data collection included demographics (age, sex, level of education, employment status, stroke/TIA diagnosis, and living situation) and assessment of level of stroke-disability according to Modified Rankin Scale; ranging from 0 to 5, where 0 indicates no disability and 5 indicates severe disability [26].

Digital questionnaires were administered via the STAAR app to assess functioning. Self-efficacy for exercise was assessed by the 9-item Exercise Self-Efficacy Scale [27]. For each item, participants indicate their confidence to execute the behavior on a 100-point percentage scale divided into 10-point increments, ranging from 0% (not at all confident) to 100% (highly confident). Fatigue was assessed by the Fatigue Severity Scale 9-item version scored on a 7-point Likert scale, ranging from 1 (“disagree”) to 7 (“fully agree”), with the median score of the 9 items used for analysis [28]. Levels of depression, anxiety, and stress were assessed by the Depression, Anxiety, and Stress Scale 21 [29]. Each of the 21 items is rated on a 4-point Likert scale ranging from 0 (“did not apply to me at all”) to 3 (“applied to me very much, or most of the time”), 7 items each pertaining to depression, anxiety, and stress. Scores for the subscales were calculated by summing the scores of each domain. Self-perceived impact of stroke was assessed using the 8 subscales (strength, memory, emotion, communication, activities of daily living/instrumental activities of daily living, mobility, hand function, social and participation) of the Stroke Impact Scale [30]. The score ranges from 0 to 100; higher scores indicate a lower perceived impact. Recovery after stroke was assessed with a visual analog scale, in which participants were asked to score their global perceived stroke recovery from 0 (“no recovery”) to 100 (“complete recovery”) [30].

#### Measures of Adherence to Supervised Exercise and Individual Counseling Sessions

Adherence to supervised exercise and individual counseling sessions during the intervention was documented in the digital clinic by the physical therapist. The intended adherence was 2 supervised exercise sessions per week in phase 1 and one exercise session per week in phase 2, along with eight counseling sessions across phases 1 and 2. Individual adherence rates in percentage were calculated by dividing exercise sessions attended by the total sessions offered in phase 1 (n=22) and phase 2 (n=12). The same approach was applied to calculate adherence to the counseling sessions offered (n=8). Based on their adherence to supervised exercise, participants were categorized into high adherence (>80%) and low adherence (<80%) groups [22].

#### App Engagement Measures

Participants’ weekly app engagement was tracked via user logs and assessed during the intervention and postintervention, with an “active week” defined as at least one interaction with the chat function (only intervention period), prescribed exercises, or activity diary. Overall app engagement was then calculated as the percentage of active weeks over the 48-week study period, excluding the introductory week. Based on this metric, participants were categorized into high app engagement (≥80%) and low app engagement (<80%) groups. Disengagement was defined as the point at which participants ceased interacting with self-managed mHealth support (ie, the activity diary and the prescribed exercise videos) for the remainder of the study until completion. Time to disengagement was calculated as the number of weeks from the start of the postintervention period to the participant’s last recorded engagement.

Participants were categorized by activity diary use as low (<19 wk), sporadic (20‐39 wk), or frequent (>40 wk) engagers and by engagement with prescribed exercises as limited (≤5 sessions), sporadic (6‐19 sessions), or frequent (>20 sessions) engagers. Participant categorization by engagement with activity diary and prescribed exercises was guided by a pragmatic approach, with thresholds selected based on the observed distribution of data. Engagement with educational videos across the entire study period was calculated as the number of viewed videos divided by the total number available (n=8).

#### Physical Activity Measures

To explore the relationship between intervention adherence, app engagement, and maintenance of physical activity in daily life, physical activity was measured objectively using the activPAL accelerometer (PAL Technologies Ltd) at baseline, and at 6- and 12-month postbaseline [31]. The activPAL device demonstrates reliability and validity in measuring step count [32] and is recommended for assessing physical activity after a stroke [33]. Participants received the monitor by mail with instructions to wear it on their nonaffected or dominant leg (as applicable) for 7 consecutive days (24 h/d) before returning it via prepaid post. Participants with at least 3 days of physical activity data, each containing a minimum of 10 hours of data, were included in the analysis [34]. The outcome was the number of steps per day. As no minimal clinically important difference in steps has been established for the stroke population, we used at least 7000 steps/day as a criterion for optimal cardiovascular risk reduction [35]. This cut-off was also deemed a reasonable and achievable target at the 6- and 12-month follow-ups, given that the baseline physical activity level in our sample was approximately 6000 steps per day. Changes in physical activity between baseline and the 6-month follow-up, and between the 6- and 12-month follow-up, were grouped into 4 categories:

1. Unchanged—low physical activity (<7000 steps/d at baseline and 6-mo follow-up, <7000 steps/d at 6-mo and 12-mo follow-up)
2. Declined physical activity (≥7000 steps/d at baseline and <7000 at 6-mo follow-up, >7000 steps/d at 6-mo follow-up and <7000 at 12-mo follow-up)
3. Unchanged—active (≥7000 steps/d at baseline and 6-mo follow-up, ≥7000 steps/d at 6-mo and 12-mo follow-up)
4. Improved physical activity (<7000 steps/d at baseline and ≥7000 at 6 mo follow-up, <7000 steps/d at 6 mo follow-up and ≥7000 at 12 mo follow-up).

Participants in categories 3 and 4 were classified as having maintained their level of physical activity.

### Data Analysis

IBM SPSS Statistics software (version 24.0; IBM Corp) was used for statistical analyses. Baseline characteristics and descriptive statistics for aims 1 to 3 were calculated and presented as mean (SD), median (IQR), and frequencies (%).

For the first and second aim, descriptive statistics described the adherence to and engagement in the mHealth intervention across the study period. Participants who completed the introductory week and began intervention phase 1 were included in the analysis. Those who discontinued the intervention were considered lost to follow-up, with data analyzed up to the week of discontinuation.

For the third aim, between-group differences regarding demographic characteristics between the sub-groups with high and low adherence, and high and low app engagement, were analyzed using Mann-Whitney *U* test for continuous variables and chi-square test for binary variables.

For the fourth aim, logistic regression analysis was used to examine the associations between high adherence during the intervention, high app engagement during the intervention and postintervention periods, and maintained physical activity levels (categories 3 and 4, as described earlier) from baseline to the 6-month follow-up and from the 6-month to the 12-month follow-up. These analyses included age (years) and use of a walking aid as covariates, as they have been shown to be associated with the level of physical activity in people poststroke [36]. The α level was set at .05 for all analysis.

## Results

### Characteristics of Participants

**Figure 3.**
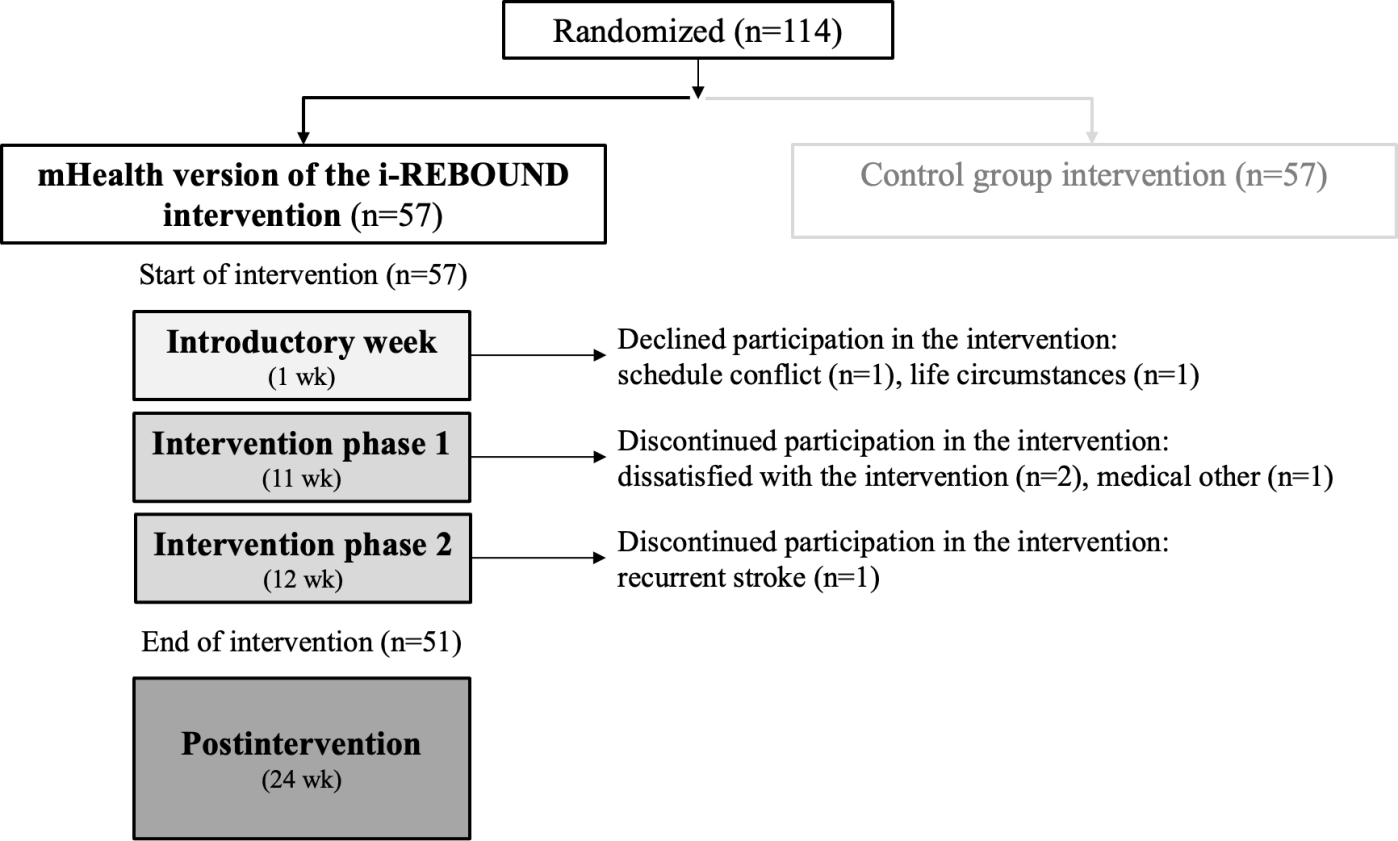
Participant flow chart.

Of the 57 participants enrolled, 51 (89%) completed the intervention (Figure 3). The mean age of the participants completing the intervention was 71 (SD 8) years, and the median time since stroke or TIA was 2.4 (IQR 1.2-5) years. Of the 51 participants who completed the intervention, 34 (67%) were female, 27 (53%) had a university degree, 8 (16%) were employed, and 47 (93%) reported no or mild poststroke or TIA symptoms (ie, Modified Rankin Scale score 0‐1) (Table 1).

**Table 1.** Baseline demographic and clinical characteristics of participants who completed the intervention versus those who discontinued, and both groups pooled.

	Completed intervention (n=51)	Discontinued (n=6)	All (n=57)
Age, mean (SD)	71 (8)	70 (12)	71 (9)
Sex, female, n (%)	34 (67)	4 (67)	38 (67)
University degree, n (%)	27 (53)	4 (67)	31 (54)
Employed, n (%)	8 (16)	1 (17)	9 (16)
TIA^a^, n (%)	15 (29)	2 (33)	17 (30)
Stroke, n (%)	36 (71)	4 (67)	40 (70)
Years since stroke, median (IQR)	2.4 (1.2-5)	2.4 (1.1-4.6)	2.4 (1.2-4.9)
Use walking aid, n (%)	16 (31)	2 (33)	18 (32)
Living with someone, n (%)	34 (67)	2 (33)	36 (63)
Modified Rankin Scale, n (%)			
No symptoms	10 (20)	—^b^	10 (18)
No significant symptoms	37 (73)	6 (100)	43 (75)
Slight disability	3 (6)	—	3 (5)
Moderate disability	1 (2)	—	1 (2)
Depression, Anxiety, and Stress Scale (0‐42), median (IQR)			
Depression	4 (2-10)	5 (4-14)	4 (2-10)
Anxiety	2 (0-6)	3 (2-8)	2 (0-6)
Stress	6 (4-10)	13 (0-19)	8 (3-10)
Stroke Impact Scale (0‐100), median (IQR)			
Strength	75 (63-94)	81 (75-95)	81 (63-94)
Memory	91 (81-94)	89 (66-98)	91 (80-95)
Emotion	53 (47-56)	53 (47-63)	53 (47-56)
Communication	96 (89-100)	96 (85-97)	96 (89-100)
ADL/iADL^c^	94 (83-98)	93 (85-97)	94 (83-98)
Mobility	88 (76-98)	89 (82-94)	88 (76-98)
Hand function	95 (65-100)	85 (78-100)	90 (70-100)
Social participation	81 (61-97)	72 (59-81)	78 (61-94)
Recovery	77 (60-90)	58 (44-83)	77 (55-90)
Self-efficacy for exercise (0‐90), median (IQR)	68 (54-79)	58 (26-77)	66 (53-79)
Fatigue Severity Scale (1-7), median (IQR)	4 (3-5)	4 (3-6)	4 (3-5)
Physical activity ≥7000 steps/d, n (%)	24 (47)	1 (17)	25 (44)

a TIA: transient ischemic attack.

b Not applicable.

c ADL/iADL: activities of daily living/instrumental activities of daily living.

### Adherence to Supervised Exercise and Individual Counseling Sessions

**Figure 4.**
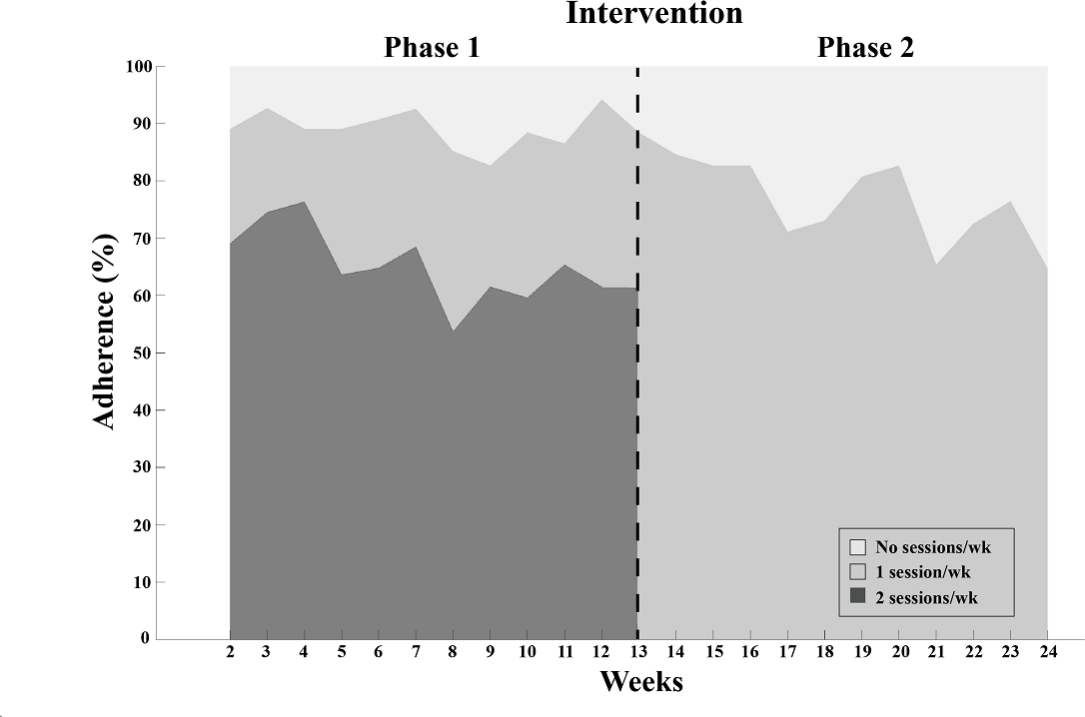
Weekly adherence to supervised exercise during intervention phases 1‐2.

The mean adherence to supervised exercise across the intervention period was 79% (min-max: 29%‐100%) (Figure 4), with 911 of 1151 sessions attended in phase 1 and 489 of 618 sessions attended in phase 2. The mean adherence rate to individual counseling sessions across the intervention was 97% (min-max: 50%‐100%) and among those who completed the intervention, 82% (42/51) attended all counseling sessions.

### Engagement With Self-Managed mHealth Support

**Figure 5.**
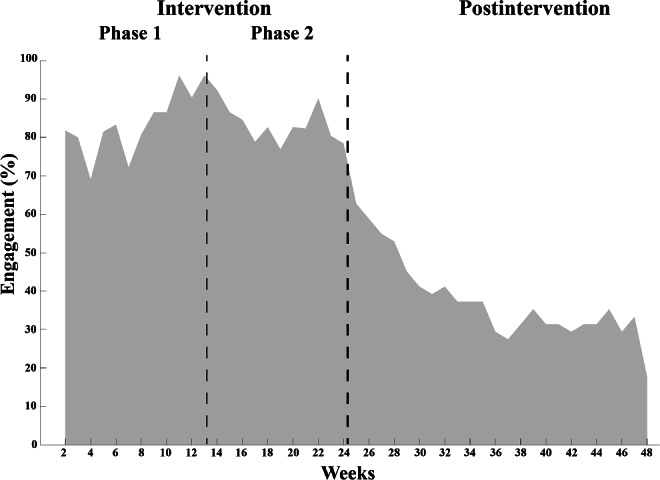
Weekly level of app engagement among the participants during the intervention and postintervention period.

App engagement was higher during the intervention phase, with an average of 84% of participants using the app weekly (min-max: 69%‐94%), compared to the postintervention period (average 38%; min-max: 18%‐63%) (Figure 5). The majority of participants (42/51, 82%) disengaged during the postintervention period, with an average time to disengagement of 8 weeks (min-max, 0‐23).

**Figure 6.**
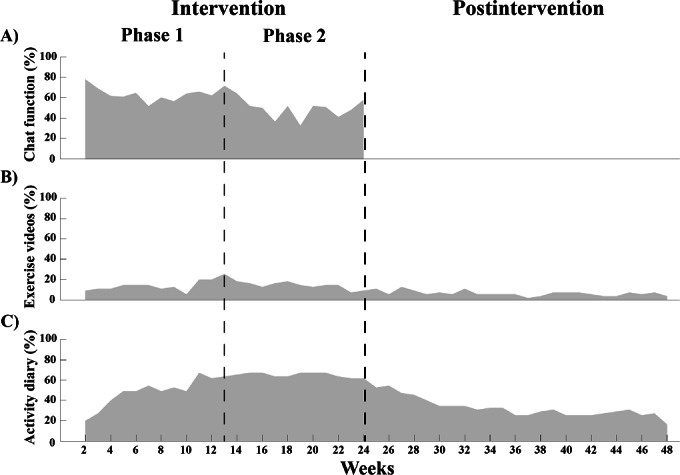
Weekly engagement in the chat function (A), exercise videos (B), and activity diary (C) during the intervention and postintervention periods.

Engagement with the chat function and with the activity diary were the most common types of app engagement (Figure 6). The average weekly engagement with the chat function was 63% in phase 1, decreasing to 50% in phase 2 (Figure 6A). On average, participants sent 1.6 weekly chat messages during the intervention. Forty-three of 55 participants (78%) used the activity diary to some extent across the study period (Figure 6C). In total, 103 activities were monitored, and the most frequently monitored activities were outdoor walking (n=29, 28%), aerobic exercise (n=13, 13%), and unspecified physical exercise (n=12, 12%). Among the 43 participants using the activity diary, 14 (33%) demonstrated low usage (<19 wk), 17 (40%) sporadic usage (20‐39 wk), and 12 (28%) frequent usage (>40 wk). Fifty of 55 participants (91%) were prescribed individual exercises, and among those, 33 (66%) had limited engagement (<5 sessions), 12 (24%) sporadic engagement (6‐19 sessions), and 5 (10%) frequent engagement (>20 sessions). The mean engagement with educational videos was 67% (min-max: 0%‐100%), with each participant viewing 5 of 8 videos on average, and 53% (29/55) participants watching all content.

### Comparison of Characteristics of Users With Different Levels of Adherence and App Engagement

Of the 51 participants completing the intervention, 31 (61%) had high adherence (ie, ≥80%) and 15 (29%) had high app engagement (ie, ≥80%). Most personal and contextual factors, as well as clinical and functional measures, showed no significant differences between participants with high versus low adherence or high versus low app engagement in the mHealth intervention (Table 2). A higher proportion of females (24/31, 77%) demonstrated high adherence to the intervention compared to males (7/31, 23%, *χ*²_1_=4.1; *P*=.04).

**Table 2.** Comparison between 4 user groups.

Variable	High adherence (n=31)	Low adherence (n=20)	*P* value	High app engagement (n=15)	Low app engagement (n=36)	*P* value
Age (years), median (IQR)	72 (68-78)	71 (64-77)	.38	72 (63-76)	72 (68-78)	.76
Sex, female, n (%)	24 (77)	10 (50)	.04	9 (60)	25 (69)	.51
University degree, n (%)	16 (52)	11 (55)	.81	7 (47)	20 (55)	.56
Employed, n (%)	5 (16)	3 (16)	.91	1 (7)	7 (19)	.25
Stroke diagnosis, n (%)	22 (71)	14 (70)	.94	9 (60)	27 (75)	.28
Years since stroke, median (IQR)	2 (1.3-5)	2.5 (0.9-4.9)	.99	3 (1.3-7.7)	2.4 (1-4.6)	.53
Use walking aid, n (%)	9 (29)	7 (35)	.65	5 (33)	11 (31)	.85
Depression, Anxiety, and Stress Scale (0‐42), median (IQR)						
Depression	4 (2-10)	4 (2-12)	.39	2 (0-10)	4 (2-10)	.10
Anxiety	2 (2-6)	3 (0-6)	.21	2 (0-6)	4 (2-6)	.83
Stress	8 (2-10)	6 (4-10)	.89	4 (2-10)	8 (4-10)	.73
Stroke Impact Scale, (0‐100), median (IQR)						
Strength	75 (63-94)	81 (58-94)	.95	88 (75-100)	75 (58-92)	.14
Memory	91 (81-94)	91 (79-96)	.73	94 (78-100)	88 (81-94)	.15
Emotion	53 (47-56)	51 (47-56)	.50	56 (53-56)	51 (47-56)	.14
Communication	96 (89-100)	95 (87-99)	.54	96 (89-100)	95 (89-99)	.62
ADL/iADL^a^	94 (83-100)	96 (80-98)	.52	98 (83-100)	93 (77-95)	.11
Mobility	85 (78-98)	89 (85-99)	.70	98 (78-100)	85 (78-95)	.15
Hand function	95 (70-100)	90 (50-100)	.60	95 (70-100)	90 (61-100)	.39
Social participation	81 (69-97)	81 (51-97)	.49	86 (67-100)	76 (59-94)	.24
Recovery	79 (65-91)	75 (51-90)	.27	81 (50-99)	76 (61-90)	.23
Self-efficacy for exercise (0‐90), median (IQR)	68 (54-81)	65 (53-79)	.69	68 (51-79)	68 (56-81)	.80
Fatigue severity scale (1-7), median (IQR)	4 (3-5)	4 (3-5)	.91	3 (2-5)	4 (3-5)	.19

a ADL/iADL: activities of daily living/instrumental activities of daily living.

### Factors Associated With Maintained Physical Activity

Valid physical activity data were available for 45 participants (88%) at 6 months and 42 participants (82%) at 12 months among those who completed the intervention. Twenty-four participants (53%) maintained their physical activity between baseline and the 6-month follow-up, whereas 19 (45%) maintained their physical activity between the 6- and 12-month follow-ups (Table 3). High adherence during the intervention was associated with maintained physical activity between baseline and the 6 months follow-up (Odds ratio [OR] 12.07, 95% CI 2-72.76; *P*=.01), and high app engagement during the postintervention was associated with maintained physical activity between 6 and 12 months (OR 5.10, 95% CI 1.02-25.52; *P*=.05). High app engagement during the intervention was not associated with maintained physical activity between baseline and the 6-month follow-up (OR 2.80, 95% CI 0.55-14.28; *P*=.22), nor was high adherence during the intervention associated with maintained physical activity between the 6- and 12-month follow-ups (OR 3.53, 95% CI 0.68-18.35; *P*=.13).

**Table 3.** Study participants demonstrating ≥7000 steps/day at 6- and 12-month follow-up, as well as a classification of the change between baseline and the follow-ups.

	Classification of change^a^	
	Baseline to 6-month follow-up (n=45), n (%)	6- to 12-month follow-up (n=42), n (%)
Unchanged—low physical activity	16 (36)	16 (38)
Declined physical activity	5 (11)	7 (17)
Unchanged—physically active	17 (38)	17 (40)
Improved physical activity	7 (15)	2 (5)

a Unchanged—low physical activity (ie, <7000 steps/d at baseline and 6-mo follow-up, <7000 steps/day at 6-mo and 12-mo follow-up); declined physical activity (ie, >7000 steps/day at baseline and <7000 at 6-mo follow-up, >7000 steps/d at 6-mo follow-up and <7000 at 12-mo follow-up); unchanged—active (ie, ≥7000 steps/d at baseline and 6-mo follow-up, ≥7000 steps/d at 6-mo and 12-mo follow-up); and improved physical activity (ie, <7000 steps/d at baseline and ≥7000 at 6 mo follow-up, <7000 steps/d at 6 mo follow-up and ≥7000 at 12 mo follow-up).

## Discussion

### Principal Findings

This study investigated adherence and app engagement during and 6 months after an mHealth intervention for people poststroke or TIA. Adherence to supervised sessions remained high (>75%) across the intervention, and more females adhered to the supervised exercise than males. Engagement with self-managed mHealth support was initially high but decreased postintervention. High adherence during the intervention was associated with maintained physical activity between baseline and the 6-month follow-up, while high postintervention engagement was associated with maintained physical activity between the 6- and 12-month follow-ups.

### Findings in Context

Adherence to supervised exercise during the intervention averaged 79%, which is comparable to adherence rates in nondigital [37] and digital interventions for people poststroke or TIA [20,38]. Adherence to counseling sessions (97%) was higher compared to a previous study showing decreasing adherence to long-term nondigital physical activity support in people poststroke [39]. The current study’s digital format provided an accessible and flexible solution that may have contributed to the high adherence rates. High adherence to supervised phases of exercise programs is common [40]. In this study, weekly interactions with a physical therapist as part of the mHealth intervention likely contributed positively to adherence. Additionally, most participants attended group exercise sessions, which may have further promoted adherence by creating a sense of accountability and commitment to the group [12,38]. Low app engagement within physical activity apps is a well-known challenge in mHealth interventions [41], yet our findings showed high engagement (84%) with self-managed support modules during the intervention, particularly the chat function and activity diary. However, app engagement postintervention decreased to 38%. As with adherence, app engagement during the intervention was likely promoted by regular interaction with a physical therapist, whereas the lack of such contact postintervention may have contributed to the decreasing patterns of engagement [19,42]. Similar patterns of declining engagement have been observed in unsupervised mHealth interventions for people with chronic conditions [43]. While the supervised sessions followed a structured schedule, engagement with the self-managed mHealth support was optional and guided by the participant’s preferences. A recent review suggests that clear guidance on the frequency and type of mHealth engagement can promote maintained participant engagement [43]. Thus, the lack of explicitly defined goals of app engagement in the current mHealth intervention might further explain the decreasing app engagement postintervention.

In line with a meta-analysis of digital physical activity interventions in adults [16], our results suggest that adherence during the intervention and app engagement postintervention is important for maintaining physical activity after the intervention period. This study found no significant differences in high adherence or app engagement between sub-groups, such as age or education level, which has previously been shown to influence mHealth intervention adherence [15]. However, our results suggest that females may have adhered to supervised sessions more than males, potentially because females tend to be more proactive and engaged in health-related issues [44]. The results from this study support that adherence to and engagement with an mHealth intervention might be important factors influencing the intervention’s impact on maintained engagement in physical activity.

Several aspects of the self-managed mHealth support could be improved to enhance the app engagement in the current mHealth intervention, as well as long-term engagement in physical activity beyond the intervention period. Personalized app content commonly benefits app engagement [41]. However, despite access to an activity diary with self-selected activities and individually prescribed exercises, only 28% (12/43) and 10% (5/50), respectively, frequently engaged with these modules in the present study. Engaging mHealth features, such as prompts, gamification, and regular content updates, have been shown to significantly enhance app engagement [19,41,42]. These elements were not incorporated into the current intervention; however, integrating them in future development could be a valuable strategy to encourage maintained app engagement among individuals poststroke or TIA. Another promising approach to enhance engagement in people poststroke or TIA, particularly those with lower digital health literacy, is to develop strategies that actively involve caregivers in participating in the mHealth intervention and mediating treatment delivery. This approach has been tested in on-site interventions [45] but has been rarely implemented in digital formats in stroke trials. Furthermore, a potential refinement of the intervention design could be development of distinct phases, each with specific goals for supporting behavior change. This might include an initiation phase focusing on establishing routines for exercise and physical activity (like the present intervention), followed by a maintenance phase in which therapist support occurs less frequently. This design could be especially important for people poststroke or TIA, who often require long-term support to establish and maintain behavior change [39]. Advancing toward more independent self-management of physical activity would also require extending the intervention’s focus to support participants in developing strategies for sustaining behavior change after the intervention, which should be embedded within the individual counseling and educational materials. We believe that refinement of the mHealth version of the i-REBOUND intervention should be cocreated with people poststroke or TIA and target multiple domains, including technology, intervention design, and the establishment of clear expectations for adherence and engagement [46].

### Limitations

The findings should be interpreted cautiously given the relatively small sample size. In addition, the participants were mostly female, highly educated, and with mild stroke symptoms, which limits the generalizability of the findings to other stroke or TIA populations [47]. Furthermore, the 7000 steps/day cut-off for physical activity, while clinically relevant for optimal cardiovascular risk reduction [35], is a crude measure for detecting change over time. Moreover, participants in the sample exhibited a notably high level of physical activity at baseline, exceeding the average daily step count of 4078 for individuals with stroke [6]. User logs yielded an in-depth examination of the participants’ mHealth engagement behavior but could not sufficiently capture the multifaceted nature of engagement. For a more comprehensive understanding of engagement, exploration of participants’ subjective experiences is required, which we acknowledge as a limitation of the present study. A strength was the 48-week study period, which allowed for a thorough investigation of changes in adherence and engagement patterns over time. However, adherence and engagement levels were classified solely as ‘low‘ (<80%) or ‘high‘ (>80%). For more substantiated levels, future studies should determine adequate levels for effective mHealth engagement and adherence and set predefined benchmarks [43].

### Conclusion

Supervised mHealth support was well received, with high adherence. In contrast, the self-management modules for physical activity faced challenges in engaging participants. High adherence during the intervention was associated with physical activity maintenance immediately afterward, while high app engagement postintervention was linked to maintenance at the 12-month follow-up. These exploratory findings may be hypothesis-generating and inform future research. To enhance engagement with mHealth interventions and support maintained physical activity after the supervised phase, future studies could benefit from qualitative and cocreative approaches to better understand and refine self-managed mHealth support for individuals poststroke or TIA.

## Supplementary material

10.2196/75662 Multimedia Appendix 1 Intervention components and their alignment to the behavior change technique taxonomy (v1) by Michie et al [25].

10.2196/75662 Checklist 1 CONSORT-EHEALTH checklist (version 1.6.1).

## Abbreviations

CONSORT-EHEALTH: Consolidated Standards of Reporting Trials of Electronic and Mobile Health Applications and Online Telehealth
mHealth: mobile health
OR: odds ratio
STAAR: Stroke Treatment Through Active and Accessible Rehabilitation
TIA: transient ischemic attack
