# Supplementary material for: Adherence to and Engagement With an mHealth Physical Activity Intervention After Mild Stroke or Transient Ischemic Attack: Secondary Analysis of a Feasibility Randomized Controlled Trial
Source: JMIR Mhealth Uhealth. 2026 Mar 17;14:e75662. doi: 10.2196/75662 (PMC12994760; doi:10.2196/75662)
Supplement: Multimedia Appendix 1 [file mhealth-v14-e75662-s001.docx]

This is a Multimedia Appendix to a full manuscript published in the JMIR mHealth and uHealth.

**Intervention components and their alignment to the behavior change technique taxonomy (v1) by Michie et al. [1]:**

| **Intervention component** | **Behavior change technique corresponding the Behavior change technique taxonomy (v1) [1]** |
| --- | --- |
| *Supervised mHealth support* | |
| Supervised exercise | 2.2 Feedback on behaviour  4.1 Instruction on how to perform the behaviour  6.1 Demonstration of the behaviour |
| Individual counseling | 1.2 Problem solving  4.1 Instruction on how to perform the behaviour  5.1 Information about health consequences  8.6 Generalization of target behaviour  8.7 Graded task  9.1 Credible source |
| Goal setting | 1.1 Goal setting (behaviour)  1.3 Goal setting (outcome)  1.4 Action planning  1.5 Review of behaviour goal(s)  1.7 Review of outcome goal(s) |
| *Self-managed mHealth support* | |
| Activity diary | 2.3 Self-monitoring of behaviour  7.1 Prompt and cues |
| Educational videos | 5.1 Information about health consequences  9.1 Credible source |
| Individual pre-recorded exercise videos | 4.1 Instruction on how to perform the behaviour  6.1 Demonstration of the behaviour |

**Reference:**

1. Michie S, Richardson M, Johnston M, Abraham C, Francis J, Hardeman W, et al. The behavior change technique taxonomy (v1) of 93 hierarchically clustered techniques: building an international consensus for the reporting of behavior change interventions. Ann Behav Med. 2013 Aug;46(1):81-95. PMID: 23512568. doi: 10.1007/s12160-013-9486-6.
