# Supplementary material for: Adherence to and Engagement With an mHealth Physical Activity Intervention After Mild Stroke or Transient Ischemic Attack: Secondary Analysis of a Feasibility Randomized Controlled Trial
Source: JMIR Mhealth Uhealth. 2026 Mar 17;14:e75662. doi: 10.2196/75662 (PMC12994760; doi:10.2196/75662)
Supplement: Checklist 1 [file mhealth-v14-e75662-s002.pdf]

CONSORT-EHEALTH (V 1.6.1) - Submission/Publication Form

The CONSORT-EHEALTH checklist is intended for authors of randomized trials evaluating web-based and Internet-based applications/interventions, including mobile interventions, electronic games (incl multiplayer games), social media, certain telehealth applications, and other interactive and/or networked electronic applications. Some of the items (e.g. all subitems under item 5 - description of the intervention) may also be applicable for other study designs.

The goal of the CONSORT EHEALTH checklist and guideline is to be
a) a guide for reporting for authors of RCTs,
b) to form a basis for appraisal of an ehealth trial (in terms of validity)

CONSORT-EHEALTH items/subitems are MANDATORY reporting items for studies published in the Journal of Medical Internet Research and other journals / scientific societies endorsing the checklist.

Items numbered 1., 2., 3., 4a., 4b etc are original CONSORT or CONSORT-NPT (non-pharmacologic treatment) items.
Items with Roman numerals (i., ii, iii, iv etc.) are CONSORT-EHEALTH extensions/clarifications.

As the CONSORT-EHEALTH checklist is still considered in a formative stage, we would ask that you also RATE ON A SCALE OF 1-5 how important/useful you feel each item is FOR THE PURPOSE OF THE CHECKLIST and reporting guideline (optional).

Mandatory reporting items are marked with a red *.
In the textboxes, either copy & paste the relevant sections from your manuscript into this form - please include any quotes from your manuscript in QUOTATION MARKS,
or answer directly by providing additional information not in the manuscript, or elaborating on why the item was not relevant for this study.

YOUR ANSWERS WILL BE PUBLISHED AS A SUPPLEMENTARY FILE TO YOUR PUBLICATION IN JMIR AND ARE CONSIDERED PART OF YOUR PUBLICATION (IF ACCEPTED).
Please fill in these questions diligently. Information will not be copyedited, so please use proper spelling and grammar, use correct capitalization, and avoid abbreviations.

DO NOT FORGET TO SAVE AS PDF _AND_ CLICK THE SUBMIT BUTTON SO YOUR ANSWERS ARE IN OUR DATABASE !!!

Citation Suggestion (if you append the pdf as Appendix we suggest to cite this paper in the caption):
Eysenbach G, CONSORT-EHEALTH Group
CONSORT-EHEALTH: Improving and Standardizing Evaluation Reports of Web-based and Mobile Health Interventions
J Med Internet Res 2011;13(4):e126

URL: http://www.jmir.org/2011/4/e126/
doi: 10.2196/jmir.1923
PMID: 22209829

Logga in på Google för att spara förloppet. Läs mer

* Anger obligatorisk fråga

Your name *

First Last

Hanna Lagerlund

Primary Affiliation (short), City, Country *

University of Toronto, Toronto, Canada

Karolinska Institutet, Stockholm, Sweden

Your e-mail address *

abc@gmail.com

hanna.lagerlund@ki.se

Title of your manuscript *

Provide the (draft) title of your manuscript.

Adherence to and Engagement Within an mHealth Intervention for Physical Activity After Mild Stroke or Transient Ischemic Attack: Analysis of an Experimental Arm in a Feasibility Randomized Controlled Trial

Name of your App/Software/Intervention *

If there is a short and a long/alternate name, write the short name first and add the long name in brackets.

STAAR app (Stroke Treatment through Active a

Evaluated Version (if any)

e.g. "V1", "Release 2017-03-01", "Version 2.0.27913"

It was the first version.

Language(s) *

What language is the intervention/app in? If multiple languages are available, separate by comma (e.g. "English, French")

Swedish

URL of your Intervention Website or App

e.g. a direct link to the mobile app on app in appstore (itunes, Google Play), or URL of the website. If the intervention is a DVD or hardware, you can also link to an Amazon page.

Ditt svar

URL of an image/screenshot (optional)

Ditt svar

Accessibility *

Can an enduser access the intervention presently?

- ○ access is free and open
- ○ access only for special usergroups, not open
- ○ access is open to everyone, but requires payment/subscription/in-app purchases
- ◉ app/intervention no longer accessible
- ○ Övrigt:

Primary Medical Indication/Disease/Condition *

e.g. "Stress", "Diabetes", or define the target group in brackets after the condition, e.g. "Autism (Parents of children with)", "Alzheimers (Informal Caregivers of)"

Stroke and TIA survivors

Primary Outcomes measured in trial *

comma-separated list of primary outcomes reported in the trial

This item is not applicable, This study was par

Secondary/other outcomes

Are there any other outcomes the intervention is expected to affect?

Ditt svar

Recommended "Dose" *

What do the instructions for users say on how often the app should be used?

- ○ Approximately Daily
- ○ Approximately Weekly
- ○ Approximately Monthly
- ○ Approximately Yearly
- ◉ "as needed"
- ○ Övrigt:

Approx. Percentage of Users (starters) still using the app as recommended after 3 months *

- ○ unknown / not evaluated
- ○ 0-10%
- ○ 11-20%
- ○ 21-30%
- ○ 31-40%
- ○ 41-50%
- ○ 51-60%
- ○ 61-70%
- ◉ 71%-80%
- ○ 81-90%
- ○ 91-100%
- ○ Övrigt:

Overall, was the app/intervention effective? *

- ○ yes: all primary outcomes were significantly better in intervention group vs control
- ○ partly: SOME primary outcomes were significantly better in intervention group vs control
- ○ no statistically significant difference between control and intervention
- ○ potentially harmful: control was significantly better than intervention in one or more outcomes
- ○ inconclusive: more research is needed
- ◉ Övrigt: This item is not applicable., The submitted manuscript is part of a prc

Article Preparation Status/Stage *

At which stage in your article preparation are you currently (at the time you fill in this form)

- ○ not submitted yet - in early draft status
- ○ not submitted yet - in late draft status, just before submission
- ○ submitted to a journal but not reviewed yet
- ◉ submitted to a journal and after receiving initial reviewer comments
- ○ submitted to a journal and accepted, but not published yet
- ○ published
- ○ Övrigt:

Journal *

If you already know where you will submit this paper (or if it is already submitted), please provide the journal name (if it is not JMIR, provide the journal name under "other")

- ○ not submitted yet / unclear where I will submit this
- ○ Journal of Medical Internet Research (JMIR)
- ● JMIR mHealth and UHealth
- ○ JMIR Serious Games
- ○ JMIR Mental Health
- ○ JMIR Public Health
- ○ JMIR Formative Research
- ○ Other JMIR sister journal
- ○ Övrigt:

Is this a full powered effectiveness trial or a pilot/feasibility trial? *

- ● Pilot/feasibility
- ○ Fully powered

Manuscript tracking number *

If this is a JMIR submission, please provide the manuscript tracking number under "other" (The ms tracking number can be found in the submission acknowledgement email, or when you login as author in JMIR. If the paper is already published in JMIR, then the ms tracking number is the four-digit number at the end of the DOI, to be found at the bottom of each published article in JMIR)

- ○ no ms number (yet) / not (yet) submitted to / published in JMIR
- ● Övrigt: # 75662

TITLE AND ABSTRACT

1a) TITLE: Identification as a randomized trial in the title

1a) Does your paper address CONSORT item 1a? *

I.e does the title contain the phrase "Randomized Controlled Trial"? (if not, explain the reason under "other")

- (selected) yes
- Övrigt:

1a-i) Identify the mode of delivery in the title

Identify the mode of delivery. Preferably use “web-based” and/or “mobile” and/or “electronic game” in the title. Avoid ambiguous terms like “online”, “virtual”, “interactive”. Use “Internet-based” only if Intervention includes non-web-based Internet components (e.g. email), use “computer-based” or “electronic” only if offline products are used. Use “virtual” only in the context of “virtual reality” (3-D worlds). Use “online” only in the context of “online support groups”. Complement or substitute product names with broader terms for the class of products (such as “mobile” or “smart phone” instead of “iphone”), especially if the application runs on different platforms.

	1	2	3	4	5	
subitem not at all important	○	○	○	○	○	essential

Does your paper address subitem 1a-i? *

Copy and paste relevant sections from manuscript title (include quotes in quotation marks "like this" to indicate direct quotes from your manuscript), or elaborate on this item by providing additional information not in the ms, or briefly explain why the item is not applicable/relevant for your study

"an mHealth Intervention for Physical Activity"

1a-ii) Non-web-based components or important co-interventions in title

Mention non-web-based components or important co-interventions in title, if any (e.g., “with telephone support”).

	1	2	3	4	5	
subitem not at all important	○	○	○	○	○	essential

Does your paper address subitem 1a-ii?

Copy and paste relevant sections from manuscript title (include quotes in quotation marks "like this" to indicate direct quotes from your manuscript), or elaborate on this item by providing additional information not in the ms, or briefly explain why the item is not applicable/relevant for your study

Ditt svar

1a-iii) Primary condition or target group in the title

Mention primary condition or target group in the title, if any (e.g., “for children with Type I Diabetes”) Example: A Web-based and Mobile Intervention with Telephone Support for Children with Type I Diabetes: Randomized Controlled Trial

	1	2	3	4	5	
subitem not at all important	○	○	○	○	○	essential

Does your paper address subitem 1a-iii? *

Copy and paste relevant sections from manuscript title (include quotes in quotation marks "like this" to indicate direct quotes from your manuscript), or elaborate on this item by providing additional information not in the ms, or briefly explain why the item is not applicable/relevant for your study

After Mild Stroke or Transient Ischemic Attack

1b) ABSTRACT: Structured summary of trial design, methods, results, and conclusions

NPT extension: Description of experimental treatment, comparator, care providers, centers, and blinding status.

1b-i) Key features/functionalities/components of the intervention and comparator in the METHODS section of the ABSTRACT

Mention key features/functionalities/components of the intervention and comparator in the abstract. If possible, also mention theories and principles used for designing the site. Keep in mind the needs of systematic reviewers and indexers by including important synonyms. (Note: Only report in the abstract what the main paper is reporting. If this information is missing from the main body of text, consider adding it)

	1	2	3	4	5	
subitem not at all important	○	○	○	○	○	essential

Does your paper address subitem 1b-i? *

Copy and paste relevant sections from the manuscript abstract (include quotes in quotation marks "like this" to indicate direct quotes from your manuscript), or elaborate on this item by providing additional information not in the ms, or briefly explain why the item is not applicable/relevant for your study

"In this study, a secondary analysis of data from the experimental arm of a feasibility randomized controlled trial was conducted. The experimental group received a 6-month mHealth version of the i-REBOUND intervention, which included supervised mHealth support for physical activity and behavior change, followed by a 6-month post-intervention period with access to self-managed mHealth support. The control group received mHealth consultations via video conferencing."

1b-ii) Level of human involvement in the METHODS section of the ABSTRACT

Clarify the level of human involvement in the abstract, e.g., use phrases like “fully automated” vs. “therapist/nurse/care provider/physician-assisted” (mention number and expertise of providers involved, if any). (Note: Only report in the abstract what the main paper is reporting. If this information is missing from the main body of text, consider adding it)

	1	2	3	4	5	
subitem not at all important	○	○	○	○	○	essential

Does your paper address subitem 1b-ii?

Copy and paste relevant sections from the manuscript abstract (include quotes in quotation marks "like this" to indicate direct quotes from your manuscript), or elaborate on this item by providing additional information not in the ms, or briefly explain why the item is not applicable/relevant for your study

"supervised mHealth support for physical activity and behavior change"

1b-iii) Open vs. closed, web-based (self-assessment) vs. face-to-face assessments in the METHODS section of the ABSTRACT

Mention how participants were recruited (online vs. offline), e.g., from an open access website or from a clinic or a closed online user group (closed usergroup trial), and clarify if this was a purely web-based trial, or there were face-to-face components (as part of the intervention or for assessment). Clearly say if outcomes were self-assessed through questionnaires (as common in web-based trials). Note: In traditional offline trials, an open trial (open-label trial) is a type of clinical trial in which both the researchers and participants know which treatment is being administered. To avoid confusion, use “blinded” or “unblinded” to indicated the level of blinding instead of “open”, as “open” in web-based trials usually refers to “open access” (i.e. participants can self-enrol). (Note: Only report in the abstract what the main paper is reporting. If this information is missing from the main body of text, consider adding it)

	1	2	3	4	5	
subitem not at all important	○	○	○	○	○	essential

Does your paper address subitem 1b-iii?

Copy and paste relevant sections from the manuscript abstract (include quotes in quotation marks "like this" to indicate direct quotes from your manuscript), or elaborate on this item by providing additional information not in the ms, or briefly explain why the item is not applicable/relevant for your study

Participants were recruited through advertisements at collaborating clinics, social media, patient organizations, and the webpage of Karolinska Institutet.

Baseline data collection included digital questionnaires for reporting of demographics (age, sex, level of education, employment status, stroke/TIA diagnosis, and living situation) and assessment of level of stroke-disability according to Modified Rankin Scale. Digital questionnaires were administered via the STAAR app to assess functioning (eg. fatigue and self-efficacy).

"Adherence measures included attendance at supervised exercise and counseling sessions, while app engagement was measured by weekly interactions with self-managed mHealth support during and after the intervention. Participants' level of physical activity (steps per day) was measured using accelerometers at baseline, and at 6- and 12 months post-baseline."

1b-iv) RESULTS section in abstract must contain use data

Report number of participants enrolled/assessed in each group, the use/uptake of the intervention (e.g., attrition/adherence metrics, use over time, number of logins etc.), in addition to primary/secondary outcomes. (Note: Only report in the abstract what the main paper is reporting. If this information is missing from the main body of text, consider adding it)

	1	2	3	4	5	
subitem not at all important	○	○	○	○	○	essential

Does your paper address subitem 1b-iv?

Copy and paste relevant sections from the manuscript abstract (include quotes in quotation marks "like this" to indicate direct quotes from your manuscript), or elaborate on this item by providing additional information not in the ms, or briefly explain why the item is not applicable/relevant for your study

"Of the 57 participants enrolled, 51 (89%) completed the intervention; the average age was 71 years, 34 (67%) were female, and 47 (92%) had mild stroke symptoms. Adherence to supervised mHealth support was high (supervised exercise sessions: 79%, counseling: 98%), while engagement with self-managed mHealth support was high during the intervention (83%) but declined post-intervention (38%). "

1b-v) CONCLUSIONS/DISCUSSION in abstract for negative trials

Conclusions/Discussions in abstract for negative trials: Discuss the primary outcome - if the trial is negative (primary outcome not changed), and the intervention was not used, discuss whether negative results are attributable to lack of uptake and discuss reasons. (Note: Only report in the abstract what the main paper is reporting. If this information is missing from the main body of text, consider adding it)

	1	2	3	4	5	
subitem not at all important	○	○	○	○	○	essential

Does your paper address subitem 1b-v?

Copy and paste relevant sections from the manuscript abstract (include quotes in quotation marks "like this" to indicate direct quotes from your manuscript), or elaborate on this item by providing additional information not in the ms, or briefly explain why the item is not applicable/relevant for your study

Ditt svar

INTRODUCTION

2a) In INTRODUCTION: Scientific background and explanation of rationale

2a-i) Problem and the type of system/solution

Describe the problem and the type of system/solution that is object of the study: intended as stand-alone intervention vs. incorporated in broader health care program? Intended for a particular patient population? Goals of the intervention, e.g., being more cost-effective to other interventions, replace or complement other solutions? (Note: Details about the intervention are provided in “Methods” under 5)

	1	2	3	4	5	
subitem not at all important	○	○	○	○	○	essential

Does your paper address subitem 2a-i? *

Copy and paste relevant sections from the manuscript (include quotes in quotation marks "like this" to indicate direct quotes from your manuscript), or elaborate on this item by providing additional information not in the ms, or briefly explain why the item is not applicable/relevant for your study

"This study builds on i-REBOUND- let´s get moving, a telehealth intervention developed in Australia to support home-based exercise and the promotion and maintenance of physical activity in people post-stroke or TIA [20]. To improve reach, i-REBOUND was developed into a fully digital mHealth intervention [21], which provides 6 months of supervised support for physical activity and behavior change, followed by a 6-month post-intervention period with access to self-managed mHealth support. Effective mHealth interventions depend on individuals’ adherence and app engagement to ensure sufficient exposure to the intervention and, ultimately, to achieving the health outcomes. Thus, optimizing and tailoring support for future mHealth interventions requires a deeper understanding of how individuals adhere to and engage with these technologies. While a previous feasibility trial demonstrated that the i-REBOUND intervention was safe, acceptable, and accessible for people post-stroke or TIA across Sweden [22], it did not assess participant adherence or engagement over time, nor the association with physical activity outcomes. Therefore, the present secondary analysis examines patterns of adherence to and engagement with the mHealth intervention, and the association to maintained physical activity after the intervention. "

2a-ii) Scientific background, rationale: What is known about the (type of) system

Scientific background, rationale: What is known about the (type of) system that is the object of the study (be sure to discuss the use of similar systems for other conditions/diagnoses, if appropiate), motivation for the study, i.e. what are the reasons for and what is the context for this specific study, from which stakeholder viewpoint is the study performed, potential impact of findings [2]. Briefly justify the choice of the comparator.

	1	2	3	4	5	
subitem not at all important	○	○	○	○	○	essential

Does your paper address subitem 2a-ii? *

Copy and paste relevant sections from the manuscript (include quotes in quotation marks "like this" to indicate direct quotes from your manuscript), or elaborate on this item by providing additional information not in the ms, or briefly explain why the item is not applicable/relevant for your study

"Interventions combining onsite supervised physical exercise with support for physical activity through behavior change techniques appear to be particularly effective in improving cardiovascular health (e.g. systolic blood pressure) [7]. However, barriers such as lack of access to professional support, long travel times, and inadequate public transportation limit people post-stroke or TIA from attending on-site physical activity services [8, 9]. Mobile health (mHealth), i.e. healthcare delivered via mobile devices, is a promising intervention modality for promoting physical activity post-stroke [10], offering a flexible solution with the potential to support long-term physical activity. Yet, individuals post-stroke may face challenges in using mHealth technology due to limited digital literacy and stroke-related impairments such as fatigue, lack of motor skills, or cognitive difficulties, which can hinder their ability to effectively interact with mobile devices [11]. Despite these challenges, individuals post-stroke are generally satisfied with digital services and show a willingness to engage with technology-based support, indicating that mHealth may be a feasible and acceptable option for this population [12]. In the context of secondary stroke prevention, mHealth provides an underutilized opportunity to enhance access to physical activity support and reduce the risk of secondary complications after stroke or TIA [10, 13]."

"Limited research has explored adherence to and engagement with mHealth interventions in individuals post-stroke or TIA [10], and the factors influencing these behaviors remain largely unknown. A better understanding of adherence and app engagement can inform future development of mHealth interventions to promote and maintain physical activity post-stroke or TIA."

2b) In INTRODUCTION: Specific objectives or hypotheses

Does your paper address CONSORT subitem 2b? *

Copy and paste relevant sections from the manuscript (include quotes in quotation marks "like this" to indicate direct quotes from your manuscript), or elaborate on this item by providing additional information not in the ms, or briefly explain why the item is not applicable/relevant for your study

"Specific aims were to (1) describe adherence to supervised exercise and individual counseling sessions during the intervention, (2) describe engagement with the self-managed mHealth support for physical activity during intervention and post-intervention, (3) compare the characteristics of participants with high and low levels of adherence and engagement in the mHealth intervention, (4) examine whether high adherence and app engagement were associated with maintained physical activity between baseline and the 6-month follow-up, and between the 6- and 12-month follow-up."

METHODS

3a) Description of trial design (such as parallel, factorial) including allocation ratio

Does your paper address CONSORT subitem 3a? *

Copy and paste relevant sections from the manuscript (include quotes in quotation marks "like this" to indicate direct quotes from your manuscript), or elaborate on this item by providing additional information not in the ms, or briefly explain why the item is not applicable/relevant for your study

"The present study was a preplanned secondary analysis of data from the experimental arm of a feasibility randomized controlled trial (ClinicalTrials: NCT0511195). The trial included people post-stroke or TIA randomized into an experimental group receiving the mHealth version of the i-REBOUND intervention or a control group receiving mHealth consultations via videoconferencing [21, 22]. The present study was a preplanned secondary analysis of data from the experimental arm of a feasibility randomized controlled trial (ClinicalTrials: NCT0511195). The trial included people post-stroke or TIA randomized into an experimental group receiving the mHealth version of the i-REBOUND intervention or a control group receiving mHealth consultations via videoconferencing [21, 22]. The analysis focused on participants´ adherence to and engagement with the mHealth intervention and was therefore limited to the experimental arm. "

Allocation ratio was 1:1. The analysis was limited to the experimental arm, therefore allocation ratio was not disclosured in the manuscript. The information is available in the study protocol (reference: Thurston C, Bezuidenhout L, Humphries S, Johansson S, von Koch L, Häger CK, Holmlund L, Sundberg CJ, Garcia-Ptacek S, Kwak L, Nilsson M, English C, Conradsson DM. Mobile health to promote physical activity in people post stroke or transient ischemic attack - study protocol for a feasibility randomised controlled trial. BMC Neurol. 2023 Mar 28;23(1):124. doi: 10.1186/s12883-023-03163-0. PMID: 36978045; PMCID: PMC10043533.)

3b) Important changes to methods after trial commencement (such as eligibility criteria), with reasons

Does your paper address CONSORT subitem 3b? *

Copy and paste relevant sections from the manuscript (include quotes in quotation marks "like this" to indicate direct quotes from your manuscript), or elaborate on this item by providing additional information not in the ms, or briefly explain why the item is not applicable/relevant for your study

No important changes were made after trial commencement.

3b-i) Bug fixes, Downtimes, Content Changes

Bug fixes, Downtimes, Content Changes: ehealth systems are often dynamic systems. A description of changes to methods therefore also includes important changes made on the intervention or comparator during the trial (e.g., major bug fixes or changes in the functionality or content) (5-iii) and other “unexpected events” that may have influenced study design such as staff changes, system failures/downtimes, etc. [2].

	1	2	3	4	5	
subitem not at all important	○	○	○	○	○	essential

Does your paper address subitem 3b-i?

Copy and paste relevant sections from the manuscript (include quotes in quotation marks "like this" to indicate direct quotes from your manuscript), or elaborate on this item by providing additional information not in the ms, or briefly explain why the item is not applicable/relevant for your study

Ditt svar

4a) Eligibility criteria for participants

Does your paper address CONSORT subitem 4a? *

Copy and paste relevant sections from the manuscript (include quotes in quotation marks "like this" to indicate direct quotes from your manuscript), or elaborate on this item by providing additional information not in the ms, or briefly explain why the item is not applicable/relevant for your study

"Inclusion criteria were (1) clinical diagnosis of stroke or TIA between 3 months and 10 years prior to study enrollment, confirmed by the participant's physician, (2) living at home, (3) being able to walk a short distance indoors with or without a walking device, (4) being able to use a smartphone including e-signature identification with/without the help of a relative/carer and (5) having access to a stable internet connection. Exclusion criteria were (1) already meeting the recommended physical activity levels of at least 150 min per week of moderate physical activity or at least 75 min per week of vigorous-intensity physical activity, (2) severe health conditions compromising engagement in the intervention or, (3) enrolled in a concomitant clinical trial or participating in rehabilitation (e.g. aerobic exercises) at the time point of recruitment."

4a-i) Computer / Internet literacy

Computer / Internet literacy is often an implicit “de facto” eligibility criterion - this should be explicitly clarified.

	1	2	3	4	5	
subitem not at all important	○	○	○	○	○	essential

Does your paper address subitem 4a-i?

Copy and paste relevant sections from the manuscript (include quotes in quotation marks "like this" to indicate direct quotes from your manuscript), or elaborate on this item by providing additional information not in the ms, or briefly explain why the item is not applicable/relevant for your study

This is adressed in inclusion criteria 4: “being able to use a smartphone including e-signature identification with/without the help of a relative/carer and (5) having access to a stable internet connection.”

4a-ii) Open vs. closed, web-based vs. face-to-face assessments:

Open vs. closed, web-based vs. face-to-face assessments: Mention how participants were recruited (online vs. offline), e.g., from an open access website or from a clinic, and clarify if this was a purely web-based trial, or there were face-to-face components (as part of the intervention or for assessment), i.e., to what degree got the study team to know the participant. In online-only trials, clarify if participants were quasi-anonymous and whether having multiple identities was possible or whether technical or logistical measures (e.g., cookies, email confirmation, phone calls) were used to detect/prevent these.

	1	2	3	4	5	
subitem not at all important	○	○	○	○	○	essential

Does your paper address subitem 4a-ii? *

Copy and paste relevant sections from the manuscript (include quotes in quotation marks "like this" to indicate direct quotes from your manuscript), or elaborate on this item by providing additional information not in the ms, or briefly explain why the item is not applicable/relevant for your study

“Participants were recruited through advertisements at collaborating clinics, social media, patient organizations, and the webpage of Karolinska Institutet.”

"The intervention was delivered via the STAAR app (Stroke Treatment through Active and Accessible Rehabilitation), which was managed by the medical technology company Empowered Health and available on iOS and Android devices. Participants accessed all intervention components through the app, including an overview of their individual goals, educational videos, an activity diary for self-monitoring, and pre-recorded exercise videos (Figure 2). Supervised sessions were delivered via video calls within the app, and written communication occurred through a chat function. Two physical therapists with ≥ 5 years’ experience in stroke rehabilitation delivered the intervention through a web-based digital clinic connected to the app. Baseline assessments were conducted using digital questionnaires administered via the app. "

4a-iii) Information giving during recruitment

Information given during recruitment. Specify how participants were briefed for recruitment and in the informed consent procedures (e.g., publish the informed consent documentation as appendix, see also item X26), as this information may have an effect on user self-selection, user expectation and may also bias results.

	1	2	3	4	5	
subitem not at all important	○	○	○	○	○	essential

Does your paper address subitem 4a-iii?

Copy and paste relevant sections from the manuscript (include quotes in quotation marks "like this" to indicate direct quotes from your manuscript), or elaborate on this item by providing additional information not in the ms, or briefly explain why the item is not applicable/relevant for your study

"Participation in the study was voluntary, and participants could withdraw at any time without consequence. No compensation was provided. Individuals who expressed interest received both verbal and written information before providing written informed consent. This consent also included approval for the secondary analysis conducted in the present study. A detailed description of the inclusion procedure and ethical considerations is available in the study protocol [21]."

For a detailed description of information given during recruitment, please refer to the study protocol (reference: Thurston C, Bezuidenhout L, Humphries S, Johansson S, von Koch L, Häger CK, Holmlund L, Sundberg CJ, Garcia-Ptacek S, Kwak L, Nilsson M, English C, Conradsson DM. Mobile health to promote physical activity in people post stroke or transient ischemic attack - study protocol for a feasibility randomised controlled trial. BMC Neurol. 2023 Mar 28;23(1):124. doi: 10.1186/s12883-023-03163-0. PMID: 36978045; PMCID: PMC10043533.).

4b) Settings and locations where the data were collected

Does your paper address CONSORT subitem 4b? *

Copy and paste relevant sections from the manuscript (include quotes in quotation marks "like this" to indicate direct quotes from your manuscript), or elaborate on this item by providing additional information not in the ms, or briefly explain why the item is not applicable/relevant for your study

"Baseline data collection included demographics (age, sex, level of education, employment status, stroke/TIA diagnosis, and living situation) and assessment of level of stroke-disability according to Modified Rankin Scale; ranging from 0-5, where 0 indicates no disability and 5 indicates severe disability [26]. Digital questionnaires were administered via the STAAR app to assess functioning. "

"Adherence to supervised exercise and individual counseling sessions during the intervention was documented in the digital clinic by the physical therapist. "

"Participants' weekly app engagement was tracked via user logs and assessed during the intervention and post-intervention, with an 'active week' defined as at least one interaction with the chat function (only intervention period), prescribed exercises, or activity diary. "

4b-i) Report if outcomes were (self-)assessed through online questionnaires

Clearly report if outcomes were (self-)assessed through online questionnaires (as common in web-based trials) or otherwise.

	1	2	3	4	5	
subitem not at all important	○	○	○	○	○	essential

Does your paper address subitem 4b-i? *

Copy and paste relevant sections from the manuscript (include quotes in quotation marks "like this" to indicate direct quotes from your manuscript), or elaborate on this item by providing additional information not in the ms, or briefly explain why the item is not applicable/relevant for your study

"Baseline data collection included demographics (age, sex, level of education, employment status, stroke/TIA diagnosis, and living situation) and assessment of level of stroke-disability according to Modified Rankin Scale; ranging from 0-5, where 0 indicates no disability and 5 indicates severe disability [26].
Digital questionnaires were administered via the STAAR app to assess functioning. Self-efficacy for exercise was assessed by the 9-item Exercise Self-Efficacy Scale [27]. For each item, participants indicate their confidence to execute the behavior on a 100-point percentage scale divided into 10-point increments, ranging from 0% (not at all confident) to 100% (highly confident). Fatigue was assessed by the Fatigue Severity Scale 9-item version scored on a 7-point Likert scale, ranging from 1 ('disagree') to 7 ('fully agree'), with the median score of the 9 items used for analysis [28]. Levels of depression, anxiety and stress were assessed by the Depression, Anxiety, and Stress Scale 21 [29]. Each of the 21 items are rated on a 4-point Likert scale ranging from 0 ('did not apply to me at all') to 3 ('applied to me very much, or most of the time'), 7 items each pertaining to depression, anxiety and stress. Scores for the subscales were calculated by summing the scores of each domain. Self-perceived impact of stroke was assessed using the 8 subscales (strength, memory, emotion, communication, activities of daily living/instrumental activities of daily living, mobility, hand function, social and participation) of the Stroke Impact Scale [30]. The score ranges from 0-100, higher scores indicate a lower perceived impact. Recovery after stroke was assessed with a visual analogue scale, in which participants were asked to score their global perceived stroke recovery from 0 ('no recovery') to 100 ('complete recovery') [30].
"

4b-ii) Report how institutional affiliations are displayed

Report how institutional affiliations are displayed to potential participants [on ehealth media], as affiliations with prestigious hospitals or universities may affect volunteer rates, use, and reactions with regards to an intervention.(Not a required item – describe only if this may bias results)

	1	2	3	4	5	
subitem not at all important	○	○	○	○	○	essential

Does your paper address subitem 4b-ii?

Copy and paste relevant sections from the manuscript (include quotes in quotation marks "like this" to indicate direct quotes from your manuscript), or elaborate on this item by providing additional information not in the ms, or briefly explain why the item is not applicable/relevant for your study

The Karolinska Institutet logo appeared on the interface of the app´s first page.

5) The interventions for each group with sufficient details to allow replication, including how and when they were actually administered

5-i) Mention names, credential, affiliations of the developers, sponsors, and owners

Mention names, credential, affiliations of the developers, sponsors, and owners [6] (if authors/evaluators are owners or developer of the software, this needs to be declared in a “Conflict of interest” section or mentioned elsewhere in the manuscript).

	1	2	3	4	5	
subitem not at all important	○	○	○	○	○	essential

Does your paper address subitem 5-i?

Copy and paste relevant sections from the manuscript (include quotes in quotation marks "like this" to indicate direct quotes from your manuscript), or elaborate on this item by providing additional information not in the ms, or briefly explain why the item is not applicable/relevant for your study

"The intervention was delivered via the STAAR app (Stroke Treatment through Active and Accessible Rehabilitation), which was managed by the medical technology company Empowered Health and available on iOS and Android devices. "

5-ii) Describe the history/development process

Describe the history/development process of the application and previous formative evaluations (e.g., focus groups, usability testing), as these will have an impact on adoption/use rates and help with interpreting results.

	1	2	3	4	5	
subitem not at all important	○	○	○	○	○	essential

Does your paper address subitem 5-ii?

Copy and paste relevant sections from the manuscript (include quotes in quotation marks "like this" to indicate direct quotes from your manuscript), or elaborate on this item by providing additional information not in the ms, or briefly explain why the item is not applicable/relevant for your study

"This study builds on i-REBOUND- let´s get moving, a telehealth intervention developed in Australia to support home-based exercise and the promotion and maintenance of physical activity in people post-stroke or TIA [20]. To improve reach, i-REBOUND was developed into a fully digital mHealth intervention [21], "

5-iii) Revisions and updating

Revisions and updating. Clearly mention the date and/or version number of the application/intervention (and comparator, if applicable) evaluated, or describe whether the intervention underwent major changes during the evaluation process, or whether the development and/or content was “frozen” during the trial. Describe dynamic components such as news feeds or changing content which may have an impact on the replicability of the intervention (for unexpected events see item 3b).

	1	2	3	4	5	
subitem not at all important	○	○	○	○	○	essential

Does your paper address subitem 5-iii?

Copy and paste relevant sections from the manuscript (include quotes in quotation marks "like this" to indicate direct quotes from your manuscript), or elaborate on this item by providing additional information not in the ms, or briefly explain why the item is not applicable/relevant for your study

It is the first version of the STAAR app. The development and/or content was “frozen” during the trial.

5-iv) Quality assurance methods

Provide information on quality assurance methods to ensure accuracy and quality of information provided [1], if applicable.

	1	2	3	4	5	
subitem not at all important	○	○	○	○	○	essential

Does your paper address subitem 5-iv?

Copy and paste relevant sections from the manuscript (include quotes in quotation marks "like this" to indicate direct quotes from your manuscript), or elaborate on this item by providing additional information not in the ms, or briefly explain why the item is not applicable/relevant for your study

Ditt svar

5-v) Ensure replicability by publishing the source code, and/or providing screenshots/screen-capture video, and/or providing flowcharts of the algorithms used

Ensure replicability by publishing the source code, and/or providing screenshots/screen-capture video, and/or providing flowcharts of the algorithms used. Replicability (i.e., other researchers should in principle be able to replicate the study) is a hallmark of scientific reporting.

	1	2	3	4	5	
subitem not at all important	○	○	○	○	○	essential

Does your paper address subitem 5-v?

Copy and paste relevant sections from the manuscript (include quotes in quotation marks "like this" to indicate direct quotes from your manuscript), or elaborate on this item by providing additional information not in the ms, or briefly explain why the item is not applicable/relevant for your study

Screenshots are provided in Figure 2 in the manuscript.

5-vi) Digital preservation

Digital preservation: Provide the URL of the application, but as the intervention is likely to change or disappear over the course of the years; also make sure the intervention is archived (Internet Archive, webcitation.org, and/or publishing the source code or screenshots/videos alongside the article). As pages behind login screens cannot be archived, consider creating demo pages which are accessible without login.

	1	2	3	4	5	
subitem not at all important	○	○	○	○	○	essential

Does your paper address subitem 5-vi?

Copy and paste relevant sections from the manuscript (include quotes in quotation marks "like this" to indicate direct quotes from your manuscript), or elaborate on this item by providing additional information not in the ms, or briefly explain why the item is not applicable/relevant for your study

Ditt svar

5-vii) Access

Access: Describe how participants accessed the application, in what setting/context, if they had to pay (or were paid) or not, whether they had to be a member of specific group. If known, describe how participants obtained “access to the platform and Internet” [1]. To ensure access for editors/reviewers/readers, consider to provide a “backdoor” login account or demo mode for reviewers/readers to explore the application (also important for archiving purposes, see vi).

	1	2	3	4	5	
subitem not at all important	○	○	○	○	○	essential

Does your paper address subitem 5-vii? *

Copy and paste relevant sections from the manuscript (include quotes in quotation marks "like this" to indicate direct quotes from your manuscript), or elaborate on this item by providing additional information not in the ms, or briefly explain why the item is not applicable/relevant for your study

"The mHealth intervention provided support for physical activity and behavior change to people post-stroke or TIA across 48 weeks divided into an intervention and post-intervention period (Figure 1). Full intervention details are outlined in the study protocol [21]. The intervention was delivered via the STAAR app (Stroke Treatment through Active and Accessible Rehabilitation), which was managed by the medical technology company Empowered Health and available on iOS and Android devices. Participants accessed all intervention components through the app, including an overview of their individual goals, educational videos, an activity diary for self-monitoring, and pre-recorded exercise videos (Figure 2). Supervised sessions were delivered via video calls within the app, and written communication occurred through a chat function. Two physical therapists with ≥ 5 years' experience in stroke rehabilitation delivered the intervention through a web-based digital clinic connected to the app. " The participants did not pay for accessing the app.

5-viii) Mode of delivery, features/functionalities/components of the intervention and comparator, and the theoretical framework

Describe mode of delivery, features/functionalities/components of the intervention and comparator, and the theoretical framework [6] used to design them (instructional strategy [1], behaviour change techniques, persuasive features, etc., see e.g., [7, 8] for terminology). This includes an in-depth description of the content (including where it is coming from and who developed it) [1],” whether [and how] it is tailored to individual circumstances and allows users to track their progress and receive feedback” [6]. This also includes a description of communication delivery channels and – if computer-mediated communication is a component – whether communication was synchronous or asynchronous [6]. It also includes information on presentation strategies [1], including page design principles, average amount of text on pages, presence of hyperlinks to other resources, etc. [1].

	1	2	3	4	5	
subitem not at all important	○	○	○	○	○	essential

?

Does your paper address subitem 5-viii? *

Copy and paste relevant sections from the manuscript (include quotes in quotation marks "like this" to indicate direct quotes from your manuscript), or elaborate on this item by providing additional information not in the ms, or briefly explain why the item is not applicable/relevant for your study

In Figure 2 in the manuscript an overview of the mHealth intervention components and the STAAR app´s interface is provided.

"Supervised mHealth support
During the first week, the mHealth intervention was introduced in 2 sessions with the physical therapist, including a medical history review, discussions about resources for physical activity and home exercises, and a practice exercise session. Phase 1 of the intervention (weeks 2-12) offered 2 supervised exercise sessions per week, and phase 2 (weeks 13-24) offered 1 session. The supervised 30-minute exercise sessions aimed to reach physical activity at moderate intensity (i.e. Borg Rating of Perceived Exercise Exertion Scale >12 [24]) and were delivered individually or in groups based on participants' physical capacity.
Support for behavior change consisted of individual monthly counseling sessions with the physical therapist. These sessions included discussions on goals, self-management strategies, and barriers and facilitators to physical activity. In addition to the supervised exercise sessions, the participants were encouraged to engage in any physical activity of their choice. The final counseling session during the last week of the intervention period focused on developing an individualized self-management plan and setting goals for the post-intervention period, supporting maintenance of exercise routines and engagement in physical activity.
Self-managed mHealth support
Educational videos on physical activity and exercise recommendations, individually prescribed exercises videos, and an activity diary were available throughout the intervention and post-intervention period as behavioral change techniques to support the promotion of physical activity in analogy with the behavior change technique taxonomy (v1) by Michie et al. [25] (see Multimedia appendix 2). Participants were encouraged to engage with the self-managed mHealth support across the intervention and post-intervention periods, although no specific instructions for intended usage were provided. Individual exercises, with pre-recorded instructional videos for strength and/or aerobic training, were prescribed by the physical therapist based on the needs and preferences of each participant. The digital activity diary encouraged self-management by enabling participants to self-monitor their physical activity. Participants selected activities to monitor, such as outdoor walking, and designated days to perform them, marking each activity as ‘performed’ or ‘not performed’ in the diary. The participants had the option to receive mobile phone reminders for the selected activities through the activity diary. During post-intervention, the study participants had access to self-managed mHealth support, but no physical therapist contact was offered.
"

5-ix) Describe use parameters

Describe use parameters (e.g., intended “doses” and optimal timing for use). Clarify what instructions or recommendations were given to the user, e.g., regarding timing, frequency, heaviness of use, if any, or was the intervention used ad libitum.

	1	2	3	4	5	
subitem not at all important	○	○	○	○	○	essential

Does your paper address subitem 5-ix?

Copy and paste relevant sections from the manuscript (include quotes in quotation marks "like this" to indicate direct quotes from your manuscript), or elaborate on this item by providing additional information not in the ms, or briefly explain why the item is not applicable/relevant for your study

Engagement: "Participants were encouraged to engage with the self-managed mHealth support across the intervention and post-intervention periods, although no specific instructions for intended usage were provided."

Adherence:"The intended adherence was two supervised exercise sessions per week in phase 1 and one exercise session per week in phase 2, along with eight counseling sessions across phases 1 and 2."

5-x) Clarify the level of human involvement

Clarify the level of human involvement (care providers or health professionals, also technical assistance) in the e-intervention or as co-intervention (detail number and expertise of professionals involved, if any, as well as “type of assistance offered, the timing and frequency of the support, how it is initiated, and the medium by which the assistance is delivered”. It may be necessary to distinguish between the level of human involvement required for the trial, and the level of human involvement required for a routine application outside of a RCT setting (discuss under item 21 – generalizability).

	1	2	3	4	5	
subitem not at all important	○	○	○	○	○	essential

Does your paper address subitem 5-x?

Copy and paste relevant sections from the manuscript (include quotes in quotation marks "like this" to indicate direct quotes from your manuscript), or elaborate on this item by providing additional information not in the ms, or briefly explain why the item is not applicable/relevant for your study

"Two physical therapists with ≥ 5 years' experience in stroke rehabilitation delivered the intervention through a web-based digital clinic connected to the app."

"Supervised mHealth support
During the first week, the mHealth intervention was introduced in 2 sessions with the physical therapist, including a medical history review, discussions about resources for physical activity and home exercises, and a practice exercise session. Phase 1 of the intervention (weeks 2-12) offered 2 supervised exercise sessions per week, and phase 2 (weeks 13-24) offered 1 session. The supervised 30-minute exercise sessions aimed to reach physical activity at moderate intensity (i.e. Borg Rating of Perceived Exercise Exertion Scale >12 [24]) and were delivered individually or in groups based on participants' physical capacity.
Support for behavior change consisted of individual monthly counseling sessions with the physical therapist. These sessions included discussions on goals, self-management strategies, and barriers and facilitators to physical activity. In addition to the supervised exercise sessions, the participants were encouraged to engage in any physical activity of their choice. The final counseling session during the last week of the intervention period focused on developing an individualized self-management plan and setting goals for the post-intervention period, supporting maintenance of exercise routines and engagement in physical activity.
"

5-xi) Report any prompts/reminders used

Report any prompts/reminders used: Clarify if there were prompts (letters, emails, phone calls, SMS) to use the application, what triggered them, frequency etc. It may be necessary to distinguish between the level of prompts/reminders required for the trial, and the level of prompts/reminders for a routine application outside of a RCT setting (discuss under item 21 – generalizability).

	1	2	3	4	5	
subitem not at all important	○	○	○	○	○	essential

Does your paper address subitem 5-xi? *

Copy and paste relevant sections from the manuscript (include quotes in quotation marks "like this" to indicate direct quotes from your manuscript), or elaborate on this item by providing additional information not in the ms, or briefly explain why the item is not applicable/relevant for your study

"The participants had the option to receive mobile phone reminders for the selected activities through the activity diary."

5-xii) Describe any co-interventions (incl. training/support)

Describe any co-interventions (incl. training/support): Clearly state any interventions that are provided in addition to the targeted eHealth intervention, as ehealth intervention may not be designed as stand-alone intervention. This includes training sessions and support [1]. It may be necessary to distinguish between the level of training required for the trial, and the level of training for a routine application outside of a RCT setting (discuss under item 21 – generalizability.

	1	2	3	4	5	
subitem not at all important	○	○	○	○	○	essential

Does your paper address subitem 5-xii? *

Copy and paste relevant sections from the manuscript (include quotes in quotation marks "like this" to indicate direct quotes from your manuscript), or elaborate on this item by providing additional information not in the ms, or briefly explain why the item is not applicable/relevant for your study

There were no co-interventions provided.

6a) Completely defined pre-specified primary and secondary outcome measures, including how and when they were assessed

Does your paper address CONSORT subitem 6a? *

Copy and paste relevant sections from the manuscript (include quotes in quotation marks "like this" to indicate direct quotes from your manuscript), or elaborate on this item by providing additional information not in the ms, or briefly explain why the item is not applicable/relevant for your study

This item is not applicable. The submitted manuscript is part of a process evaluation, and this specific study aims to explore adherence to and engagement with an mHealth intervention (only the experimental arm was included in the analysis). It was not an efficacy trial, and therefore, primary and secondary outcomes were not defined.

6a-i) Online questionnaires: describe if they were validated for online use and apply CHERRIES items to describe how the questionnaires were designed/deployed

If outcomes were obtained through online questionnaires, describe if they were validated for online use and apply CHERRIES items to describe how the questionnaires were designed/deployed [9].

	1	2	3	4	5	
subitem not at all important	○	○	○	○	○	essential

Does your paper address subitem 6a-i?

Copy and paste relevant sections from manuscript text

We used validated questionnaires in a digital format (i.e. provided through the mobile app STAAR), however at the time of the study, the assessment scales were not validated for online use.

6a-ii) Describe whether and how “use” (including intensity of use/dosage) was defined/measured/monitored

Describe whether and how “use” (including intensity of use/dosage) was defined/measured/monitored (logins, logfile analysis, etc.). Use/adoption metrics are important process outcomes that should be reported in any ehealth trial.

	1	2	3	4	5	
subitem not at all important	○	○	○	○	○	essential

Does your paper address subitem 6a-ii?

Copy and paste relevant sections from manuscript text

"Measures of adherence to supervised exercise and individual counseling sessions
Adherence to supervised exercise and individual counseling sessions during the intervention was documented in the digital clinic by the physical therapist. The intended adherence was two supervised exercise sessions per week in phase 1 and one exercise session per week in phase 2, along with eight counseling sessions across phases 1 and 2. Individual adherence rates in percentage were calculated by dividing exercise sessions attended by the total sessions offered in phase 1 (n=22) and phase 2 (n=12). The same approach was applied to calculate adherence to the counseling sessions offered (n=8). Based on their adherence to supervised exercise participants were categorized into high adherence (>80%) and low adherence (<80%) groups [22].
App engagement measures
Participants' weekly app engagement was tracked via user logs and assessed during the intervention and post-intervention, with an 'active week' defined as at least one interaction with the chat function (only intervention period), prescribed exercises, or activity diary. Overall app engagement was then calculated as the percentage of active weeks over the 48-week study period, excluding the introductory week. Based on this metric, participants were categorized into high app engagement (≥80%) and low app engagement <80%) groups. Disengagement was defined as the point at which participants ceased interacting with self-managed mHealth support (i.e. the activity diary and the prescribed exercise videos) for the remainder of the study until completion. Time to disengagement was calculated as the number of weeks from the start of the post-intervention period to the participant's last recorded engagement.
Participants were categorized by activity diary use as low (<19 weeks), sporadic (20–39 weeks), or frequent (>40 weeks) engagers and by engagement with prescribed exercises as limited (≤5 sessions), sporadic (6–19 sessions), or frequent (>20 sessions) engagers. Participant categorization by engagement with activity diary and prescribed exercises was guided by a pragmatic approach, with thresholds selected based on the observed distribution of data. Engagement with educational videos across the entire study period was calculated as the number of viewed videos divided by the total number available (n=8).
"

6a-iii) Describe whether, how, and when qualitative feedback from participants was obtained

Describe whether, how, and when qualitative feedback from participants was obtained (e.g., through emails, feedback forms, interviews, focus groups).

	1	2	3	4	5	
subitem not at all important	○	○	○	○	○	essential

Does your paper address subitem 6a-iii?

Copy and paste relevant sections from manuscript text

We have conducted two qualitative studies (both currently in manuscript format). These studies offer valuable perspectives that complement the adherence and app engagement data; however, their results are beyond the scope of the current manuscript. This omission is acknowledged as a limitation and is addressed in the following statement in the discussion section with the following quote: “For a more comprehensive understanding of engagement, exploration of participants’ subjective experiences is required, which we acknowledge as a limitation of the present study. “

6b) Any changes to trial outcomes after the trial commenced, with reasons

Does your paper address CONSORT subitem 6b? *

Copy and paste relevant sections from the manuscript (include quotes in quotation marks "like this" to indicate direct quotes from your manuscript), or elaborate on this item by providing additional information not in the ms, or briefly explain why the item is not applicable/relevant for your study

No, there were no changes made.

7a) How sample size was determined

NPT: When applicable, details of whether and how the clustering by care provides or centers was addressed

7a-i) Describe whether and how expected attrition was taken into account when calculating the sample size

Describe whether and how expected attrition was taken into account when calculating the sample size.

	1	2	3	4	5	
subitem not at all important	○	○	○	○	○	essential

Does your paper address subitem 7a-i?

Copy and paste relevant sections from manuscript title (include quotes in quotation marks "like this" to indicate direct quotes from your manuscript), or elaborate on this item by providing additional information not in the ms, or briefly explain why the item is not applicable/relevant for your study

The sample size is in line with recommendations regarding feasibility studies. For detailed information on sample size calculations, please refer to the study protocol (reference: Thurston C, Bezuidenhout L, Humphries S, Johansson S, von Koch L, Häger CK, Holmlund L, Sundberg CJ, Garcia-Ptacek S, Kwak L, Nilsson M, English C, Conradsson DM. Mobile health to promote physical activity in people post stroke or transient ischemic attack - study protocol for a feasibility randomised controlled trial. BMC Neurol. 2023 Mar 28;23(1):124. doi: 10.1186/s12883-023-03163-0. PMID: 36978045; PMCID: PMC10043533.).

7b) When applicable, explanation of any interim analyses and stopping guidelines

Does your paper address CONSORT subitem 7b? *

Copy and paste relevant sections from the manuscript (include quotes in quotation marks "like this" to indicate direct quotes from your manuscript), or elaborate on this item by providing additional information not in the ms, or briefly explain why the item is not applicable/relevant for your study

This item is not applicable.

8a) Method used to generate the random allocation sequence

NPT: When applicable, how care providers were allocated to each trial group

Does your paper address CONSORT subitem 8a? *

Copy and paste relevant sections from the manuscript (include quotes in quotation marks "like this" to indicate direct quotes from your manuscript), or elaborate on this item by providing additional information not in the ms, or briefly explain why the item is not applicable/relevant for your study

For detailed information on generation of the random allocation sequence, please refer to the study protocol, (reference: Thurston C, Bezuidenhout L, Humphries S, Johansson S, von Koch L, Häger CK, Holmlund L, Sundberg CJ, Garcia-Ptacek S, Kwak L, Nilsson M, English C, Conradsson DM. Mobile health to promote physical activity in people post stroke or transient ischemic attack - study protocol for a feasibility randomised controlled trial. BMC Neurol. 2023 Mar 28;23(1):124. doi: 10.1186/s12883-023-03163-0. PMID: 36978045; PMCID: PMC10043533.)

8b) Type of randomisation; details of any restriction (such as blocking and block size)

Does your paper address CONSORT subitem 8b? *

Copy and paste relevant sections from the manuscript (include quotes in quotation marks "like this" to indicate direct quotes from your manuscript), or elaborate on this item by providing additional information not in the ms, or briefly explain why the item is not applicable/relevant for your study

For detailed information on type of randomization, please refer to the study protocol, (reference: Thurston C, Bezuidenhout L, Humphries S, Johansson S, von Koch L, Häger CK, Holmlund L, Sundberg CJ, Garcia-Ptacek S, Kwak L, Nilsson M, English C, Conradsson DM. Mobile health to promote physical activity in people post stroke or transient ischemic attack - study protocol for a feasibility randomised controlled trial. BMC Neurol. 2023 Mar 28;23(1):124. doi: 10.1186/s12883-023-03163-0. PMID: 36978045; PMCID: PMC10043533.)

9) Mechanism used to implement the random allocation sequence (such as sequentially numbered containers), describing any steps taken to conceal the sequence until interventions were assigned

Does your paper address CONSORT subitem 9? *

Copy and paste relevant sections from the manuscript (include quotes in quotation marks "like this" to indicate direct quotes from your manuscript), or elaborate on this item by providing additional information not in the ms, or briefly explain why the item is not applicable/relevant for your study

For detailed information, please refer to the study protocol, (reference: Thurston C, Bezuidenhout L, Humphries S, Johansson S, von Koch L, Häger CK, Holmlund L, Sundberg CJ, Garcia-Ptacek S, Kwak L, Nilsson M, English C, Conradsson DM. Mobile health to promote physical activity in people post stroke or transient ischemic attack - study protocol for a feasibility randomised controlled trial. BMC Neurol. 2023 Mar 28;23(1):124. doi: 10.1186/s12883-023-03163-0. PMID: 36978045; PMCID: PMC10043533.)

10) Who generated the random allocation sequence, who enrolled participants, and who assigned participants to interventions

Does your paper address CONSORT subitem 10? *

Copy and paste relevant sections from the manuscript (include quotes in quotation marks "like this" to indicate direct quotes from your manuscript), or elaborate on this item by providing additional information not in the ms, or briefly explain why the item is not applicable/relevant for your study

For detailed information, please refer to the study protocol, (reference: Thurston C, Bezuidenhout L, Humphries S, Johansson S, von Koch L, Häger CK, Holmlund L, Sundberg CJ, Garcia-Ptacek S, Kwak L, Nilsson M, English C, Conradsson DM. Mobile health to promote physical activity in people post stroke or transient ischemic attack - study protocol for a feasibility randomised controlled trial. BMC Neurol. 2023 Mar 28;23(1):124. doi: 10.1186/s12883-023-03163-0. PMID: 36978045; PMCID: PMC10043533.)

11a) If done, who was blinded after assignment to interventions (for example, participants, care providers, those assessing outcomes) and how

NPT: Whether or not administering co-interventions were blinded to group assignment

11a-i) Specify who was blinded, and who wasn't

Specify who was blinded, and who wasn't. Usually, in web-based trials it is not possible to blind the participants [1, 3] (this should be clearly acknowledged), but it may be possible to blind outcome assessors, those doing data analysis or those administering co-interventions (if any).

	1	2	3	4	5	
subitem not at all important	○	○	○	○	○	essential

Does your paper address subitem 11a-i? *

Copy and paste relevant sections from the manuscript (include quotes in quotation marks "like this" to indicate direct quotes from your manuscript), or elaborate on this item by providing additional information not in the ms, or briefly explain why the item is not applicable/relevant for your study

There was no blinding applied.

11a-ii) Discuss e.g., whether participants knew which intervention was the "intervention of interest" and which one was the "comparator"

Informed consent procedures (4a-ii) can create biases and certain expectations - discuss e.g., whether participants knew which intervention was the "intervention of interest" and which one was the "comparator".

	1	2	3	4	5	
subitem not at all important	○	○	○	○	○	essential

Does your paper address subitem 11a-ii?

Copy and paste relevant sections from the manuscript (include quotes in quotation marks "like this" to indicate direct quotes from your manuscript), or elaborate on this item by providing additional information not in the ms, or briefly explain why the item is not applicable/relevant for your study

Ditt svar

11b) If relevant, description of the similarity of interventions
(this item is usually not relevant for ehealth trials as it refers to similarity of a placebo or sham intervention to a active medication/intervention)

Does your paper address CONSORT subitem 11b? *

Copy and paste relevant sections from the manuscript (include quotes in quotation marks "like this" to indicate direct quotes from your manuscript), or elaborate on this item by providing additional information not in the ms, or briefly explain why the item is not applicable/relevant for your study

This item is not applicable.

12a) Statistical methods used to compare groups for primary and secondary outcomes
NPT: When applicable, details of whether and how the clustering by care providers or centers was addressed

Does your paper address CONSORT subitem 12a? *

Copy and paste relevant sections from the manuscript (include quotes in quotation marks "like this" to indicate direct quotes from your manuscript), or elaborate on this item by providing additional information not in the ms, or briefly explain why the item is not applicable/relevant for your study

This item is not applicable.

12a-i) Imputation techniques to deal with attrition / missing values
Imputation techniques to deal with attrition / missing values: Not all participants will use the intervention/comparator as intended and attrition is typically high in ehealth trials. Specify how participants who did not use the application or dropped out from the trial were treated in the statistical analysis (a complete case analysis is strongly discouraged, and simple imputation techniques such as LOCF may also be problematic [4]).

	1	2	3	4	5	
subitem not at all important	○	○	○	○	○	essential

Does your paper address subitem 12a-i? *

Copy and paste relevant sections from the manuscript (include quotes in quotation marks "like this" to indicate direct quotes from your manuscript), or elaborate on this item by providing additional information not in the ms, or briefly explain why the item is not applicable/relevant for your study

This item is not applicable.

12b) Methods for additional analyses, such as subgroup analyses and adjusted analyses

Does your paper address CONSORT subitem 12b? *

Copy and paste relevant sections from the manuscript (include quotes in quotation marks "like this" to indicate direct quotes from your manuscript), or elaborate on this item by providing additional information not in the ms, or briefly explain why the item is not applicable/relevant for your study

This item is not applicable.

X26) REB/IRB Approval and Ethical Considerations [recommended as subheading under "Methods"] (not a CONSORT item)

X26-i) Comment on ethics committee approval

	1	2	3	4	5	
subitem not at all important	○	○	○	○	○	essential

Does your paper address subitem X26-i?

Copy and paste relevant sections from the manuscript (include quotes in quotation marks "like this" to indicate direct quotes from your manuscript), or elaborate on this item by providing additional information not in the ms, or briefly explain why the item is not applicable/relevant for your study

"Ethical considerations
The study was approved by the Swedish Ethical Review Authority (dnr 2020–05062 and 2021-03622). Participation in the study was voluntary, and participants could withdraw at any time without consequence. No compensation was provided. Individuals who expressed interest received both verbal and written information before providing written informed consent. This consent also included approval for the secondary analysis conducted in the present study. A detailed description of the inclusion procedure and ethical considerations is available in the study protocol [21]. All data were anonymized to ensure participant confidentiality."

x26-ii) Outline informed consent procedures

Outline informed consent procedures e.g., if consent was obtained offline or online (how? Checkbox, etc.?), and what information was provided (see 4a-ii). See [6] for some items to be included in informed consent documents.

	1	2	3	4	5	
subitem not at all important	○	○	○	○	○	essential

Does your paper address subitem X26-ii?

Copy and paste relevant sections from the manuscript (include quotes in quotation marks "like this" to indicate direct quotes from your manuscript), or elaborate on this item by providing additional information not in the ms, or briefly explain why the item is not applicable/relevant for your study

"Participation in the study was voluntary, and participants could withdraw at any time without consequence. No compensation was provided. Individuals who expressed interest received both verbal and written information before providing written informed consent. This consent also included approval for the secondary analysis conducted in the present study. A detailed description of the inclusion procedure and ethical considerations is available in the study protocol [21]. "

X26-iii) Safety and security procedures

Safety and security procedures, incl. privacy considerations, and any steps taken to reduce the likelihood or detection of harm (e.g., education and training, availability of a hotline)

	1	2	3	4	5	
subitem not at all important	○	○	○	○	○	essential

Does your paper address subitem X26-iii?

Copy and paste relevant sections from the manuscript (include quotes in quotation marks "like this" to indicate direct quotes from your manuscript), or elaborate on this item by providing additional information not in the ms, or briefly explain why the item is not applicable/relevant for your study

During the recruitment process safety aspects related to exercising at home were discussed and the participants reflected on how to deal with safety aspects, e.g. how they would act in case of a fall during exercise. During the intervention video calls were used for supervised sessions and written communication with a physical therapist was enabled through chat.

RESULTS

13a) For each group, the numbers of participants who were randomly assigned, received intended treatment, and were analysed for the primary outcome

NPT: The number of care providers or centers performing the intervention in each group and the number of patients treated by each care provider in each center

Does your paper address CONSORT subitem 13a? *

Copy and paste relevant sections from the manuscript (include quotes in quotation marks "like this" to indicate direct quotes from your manuscript), or elaborate on this item by providing additional information not in the ms, or briefly explain why the item is not applicable/relevant for your study

"Of the 57 participants enrolled, 51 (89%) completed the intervention (Figure 3). The mean age of the participants completing the intervention was 71 years (SD: 8) and the median time since stroke or TIA was 2.4 years (IQR: 1.3). "

For detailed information, please refer to Figure 3 in the manuscript.

13b) For each group, losses and exclusions after randomisation, together with reasons

Does your paper address CONSORT subitem 13b? (NOTE: Preferably, this is shown in a CONSORT flow diagram) *

Copy and paste relevant sections from the manuscript (include quotes in quotation marks "like this" to indicate direct quotes from your manuscript), or elaborate on this item by providing additional information not in the ms, or briefly explain why the item is not applicable/relevant for your study

Please refer to “Figure 3” in the manuscript.

13b-i) Attrition diagram

Strongly recommended: An attrition diagram (e.g., proportion of participants still logging in or using the intervention/comparator in each group plotted over time, similar to a survival curve) or other figures or tables demonstrating usage/dose/engagement.

	1	2	3	4	5	
subitem not at all important	○	○	○	○	○	essential

Does your paper address subitem 13b-i?

Copy and paste relevant sections from the manuscript or cite the figure number if applicable (include quotes in quotation marks "like this" to indicate direct quotes from your manuscript), or elaborate on this item by providing additional information not in the ms, or briefly explain why the item is not applicable/relevant for your study

Please refer to Figure 5-6 in the manuscript.

14a) Dates defining the periods of recruitment and follow-up

Does your paper address CONSORT subitem 14a? *

Copy and paste relevant sections from the manuscript (include quotes in quotation marks "like this" to indicate direct quotes from your manuscript), or elaborate on this item by providing additional information not in the ms, or briefly explain why the item is not applicable/relevant for your study

“The data was collected between September 2021 and December 2023. “

14a-i) Indicate if critical “secular events” fell into the study period

Indicate if critical “secular events” fell into the study period, e.g., significant changes in Internet resources available or “changes in computer hardware or Internet delivery resources”

	1	2	3	4	5	
subitem not at all important	○	○	○	○	○	essential

Does your paper address subitem 14a-i?

Copy and paste relevant sections from the manuscript (include quotes in quotation marks "like this" to indicate direct quotes from your manuscript), or elaborate on this item by providing additional information not in the ms, or briefly explain why the item is not applicable/relevant for your study

No "secular events" fell into the study period.

14b) Why the trial ended or was stopped (early)

Does your paper address CONSORT subitem 14b? *

Copy and paste relevant sections from the manuscript (include quotes in quotation marks "like this" to indicate direct quotes from your manuscript), or elaborate on this item by providing additional information not in the ms, or briefly explain why the item is not applicable/relevant for your study

This item is not applicable.

15) A table showing baseline demographic and clinical characteristics for each group

NPT: When applicable, a description of care providers (case volume, qualification, expertise, etc.) and centers (volume) in each group

Does your paper address CONSORT subitem 15? *

Copy and paste relevant sections from the manuscript (include quotes in quotation marks "like this" to indicate direct quotes from your manuscript), or elaborate on this item by providing additional information not in the ms, or briefly explain why the item is not applicable/relevant for your study

Please refer to table 1 in the manuscript.

15-i) Report demographics associated with digital divide issues

In ehealth trials it is particularly important to report demographics associated with digital divide issues, such as age, education, gender, social-economic status, computer/Internet/ehealth literacy of the participants, if known.

	1	2	3	4	5	
subitem not at all important	○	○	○	○	○	essential

Does your paper address subitem 15-i? *

Copy and paste relevant sections from the manuscript (include quotes in quotation marks "like this" to indicate direct quotes from your manuscript), or elaborate on this item by providing additional information not in the ms, or briefly explain why the item is not applicable/relevant for your study

Please refer to table 1 in the manuscript.

16) For each group, number of participants (denominator) included in each analysis and whether the analysis was by original assigned groups

16-i) Report multiple “denominators” and provide definitions

Report multiple “denominators” and provide definitions: Report N’s (and effect sizes) “across a range of study participation [and use] thresholds” [1], e.g., N exposed, N consented, N used more than x times, N used more than y weeks, N participants “used” the intervention/comparator at specific pre-defined time points of interest (in absolute and relative numbers per group). Always clearly define “use” of the intervention.

	1	2	3	4	5	
subitem not at all important	○	○	○	○	○	essential

Does your paper address subitem 16-i? *

Copy and paste relevant sections from the manuscript (include quotes in quotation marks "like this" to indicate direct quotes from your manuscript), or elaborate on this item by providing additional information not in the ms, or briefly explain why the item is not applicable/relevant for your study

"Of the 51 participants completing the intervention, 31 (61%) had high adherence (i.e. ≥80%) and 15 (29%) had high app engagement (i.e. ≥ 80%)."

"Valid physical activity data were available for 45 participants (88%) at 6 months and 42 participants (82%) at 12 months among those who completed the intervention. "

16-ii) Primary analysis should be intent-to-treat

Primary analysis should be intent-to-treat, secondary analyses could include comparing only “users”, with the appropriate caveats that this is no longer a randomized sample (see 18-i).

	1	2	3	4	5	
subitem not at all important	○	○	○	○	○	essential

Does your paper address subitem 16-ii?

Copy and paste relevant sections from the manuscript (include quotes in quotation marks "like this" to indicate direct quotes from your manuscript), or elaborate on this item by providing additional information not in the ms, or briefly explain why the item is not applicable/relevant for your study

This item is not applicable.

17a) For each primary and secondary outcome, results for each group, and the estimated effect size and its precision (such as 95% confidence interval)

Does your paper address CONSORT subitem 17a? *

Copy and paste relevant sections from the manuscript (include quotes in quotation marks "like this" to indicate direct quotes from your manuscript), or elaborate on this item by providing additional information not in the ms, or briefly explain why the item is not applicable/relevant for your study

This item is not applicable.

17a-i) Presentation of process outcomes such as metrics of use and intensity of use

In addition to primary/secondary (clinical) outcomes, the presentation of process outcomes such as metrics of use and intensity of use (dose, exposure) and their operational definitions is critical. This does not only refer to metrics of attrition (13-b) (often a binary variable), but also to more continuous exposure metrics such as “average session length”. These must be accompanied by a technical description how a metric like a “session” is defined (e.g., timeout after idle time) [1] (report under item 6a).

	1	2	3	4	5	
subitem not at all important	○	○	○	○	○	essential

Does your paper address subitem 17a-i?

Copy and paste relevant sections from the manuscript (include quotes in quotation marks "like this" to indicate direct quotes from your manuscript), or elaborate on this item by providing additional information not in the ms, or briefly explain why the item is not applicable/relevant for your study

"The mean adherence to supervised exercise across the intervention period was 79% (min-max: 29-100%) (Figure 4), with 911 of 1151 sessions attended in phase 1 and 489 of 618 sessions attended in phase 2. The mean adherence rate to individual counseling sessions across the intervention was 97% (min-max: 50-100%) and among those who completed the intervention, 82% (n=42) attended all counseling sessions. "

"App engagement was higher during the intervention phase, with an average of 84% of participants using the app weekly (min-max: 69–94%), compared to the post-intervention period (average 38%; min-max: 18–63%) (Figure 5). The majority of participants (42/51, 82%) disengaged during the post-intervention period, with an average time to disengagement of 8 weeks (min-max, 0-23)."

17b) For binary outcomes, presentation of both absolute and relative effect sizes is recommended

Does your paper address CONSORT subitem 17b? *

Copy and paste relevant sections from the manuscript (include quotes in quotation marks "like this" to indicate direct quotes from your manuscript), or elaborate on this item by providing additional information not in the ms, or briefly explain why the item is not applicable/relevant for your study

This item is not applicable.

18) Results of any other analyses performed, including subgroup analyses and adjusted analyses, distinguishing pre-specified from exploratory

Does your paper address CONSORT subitem 18? *

Copy and paste relevant sections from the manuscript (include quotes in quotation marks "like this" to indicate direct quotes from your manuscript), or elaborate on this item by providing additional information not in the ms, or briefly explain why the item is not applicable/relevant for your study

"Engagement with the chat function and activity diary were the most common types of app engagement (Figure 6). The average weekly engagement with the chat function was 63% in phase 1, decreasing to 50% in phase 2 (Figure 6A). On average, participants sent 1.6 weekly chat messages during the intervention. Forty-three participants out of 55 (78%) utilized the activity diary to some extent across the study period (Figure 6C). In total 103 activities were monitored and the most frequently monitored activities were outdoor walking (n=29, 28%), aerobic exercise (n=13, 13%) and unspecified physical exercise (n=12, 12%). Among the 43 participants using the activity diary, 14 (33%) demonstrated low usage (<19 weeks), 17 (40%) sporadic usage (20–39 weeks), and 12 (28%) frequent usage (>40 weeks). Fifty participants out of 55 (91%) were prescribed individual exercises, and among those, 33 (66%) had limited engagement (< 5 sessions), 12 (24%) sporadic engagement (6–19 sessions), and 5 (10%) frequent engagement (>20 sessions). The mean engagement with educational videos was 67% (min-max: 0-100%), with each participant viewing 5 out of 8 videos on average, and 53% (29/55) of the participants watching all the content."

"Of the 51 participants completing the intervention, 31 (61%) had high adherence (i.e. ≥80%) and 15 (29%) had high app engagement (i.e. ≥ 80%). Most personal and contextual factors, as well as clinical and functional measures, showed no significant differences between participants with high versus low adherence or high versus low app engagement in the mHealth intervention (Table 2). A higher proportion of females (24/31, 77%) demonstrated high adherence to the intervention compared to males (7/31, 23%, χ^2=4.113, df=1, P = .043)."

"Twenty-four participants (53%) maintained their physical activity between baseline and the 6-month follow-up, whereas 19 (45%) maintained their physical activity between the 6- and 12-month follow-ups (Table 3). High adherence during the intervention was associated with maintained physical activity between baseline and the 6 months follow-up (Odds ratio (OR): 12.07, 95% CI: 2.00[illegible]72.76, P = .007), and high app engagement during the post-intervention was associated with maintained physical activity between 6 and 12 months (OR: 5.10, 95% CI: 1.02[illegible]25.52, P = .047). High app engagement during the intervention was not associated with maintained physical activity between baseline and the 6-month follow-up (OR: 2.80, 95% CI: 0.55–14.28, P = .216), nor was high adherence during the intervention associated with maintained physical activity between the 6- and 12- month follow-ups (OR: 3.53, 95% CI: 0.68–18.35, P = .134)."

18-i) Subgroup analysis of comparing only users

A subgroup analysis of comparing only users is not uncommon in ehealth trials, but if done, it must be stressed that this is a self-selected sample and no longer an unbiased sample from a randomized trial (see 16-iii).

	1	2	3	4	5	
subitem not at all important	○	○	○	○	○	essential

Does your paper address subitem 18-i?

Copy and paste relevant sections from the manuscript (include quotes in quotation marks "like this" to indicate direct quotes from your manuscript), or elaborate on this item by providing additional information not in the ms, or briefly explain why the item is not applicable/relevant for your study

This item is not applicable.

19) All important harms or unintended effects in each group

(for specific guidance see CONSORT for harms)

Does your paper address CONSORT subitem 19? *

Copy and paste relevant sections from the manuscript (include quotes in quotation marks "like this" to indicate direct quotes from your manuscript), or elaborate on this item by providing additional information not in the ms, or briefly explain why the item is not applicable/relevant for your study

This is detailed and reported in another manuscript, please refer to reference: Thurston C, Humphries S, Bezuidenhout L, et al. Mobile health delivered physical activity after mild stroke or transient ischemic attack: Is it feasible and acceptable? International Journal of Stroke. 2025;0(0). doi:10.1177/17474930251315628

19-i) Include privacy breaches, technical problems

Include privacy breaches, technical problems. This does not only include physical “harm” to participants, but also incidents such as perceived or real privacy breaches [1], technical problems, and other unexpected/unintended incidents. “Unintended effects” also includes unintended positive effects [2].

	1	2	3	4	5	
subitem not at all important	○	○	○	○	○	essential

Does your paper address subitem 19-i?

Copy and paste relevant sections from the manuscript (include quotes in quotation marks "like this" to indicate direct quotes from your manuscript), or elaborate on this item by providing additional information not in the ms, or briefly explain why the item is not applicable/relevant for your study

This is detailed and reported in another manuscript, please refer to reference: Thurston C, Humphries S, Bezuidenhout L, et al. Mobile health delivered physical activity after mild stroke or transient ischemic attack: Is it feasible and acceptable? International Journal of Stroke. 2025;0(0). doi:10.1177/17474930251315628

19-ii) Include qualitative feedback from participants or observations from staff/researchers

Include qualitative feedback from participants or observations from staff/researchers, if available, on strengths and shortcomings of the application, especially if they point to unintended/unexpected effects or uses. This includes (if available) reasons for why people did or did not use the application as intended by the developers.

	1	2	3	4	5	
subitem not at all important	○	○	○	○	○	essential

Does your paper address subitem 19-ii?

Copy and paste relevant sections from the manuscript (include quotes in quotation marks "like this" to indicate direct quotes from your manuscript), or elaborate on this item by providing additional information not in the ms, or briefly explain why the item is not applicable/relevant for your study

We have conducted two qualitative studies (both currently in manuscript format). These studies offer valuable perspectives that complement the adherence and app engagement data; however, their results are beyond the scope of the current manuscript. This omission is acknowledged as a limitation and is addressed in the following statement in the discussion section with the following quote: “For a more comprehensive understanding of engagement, exploration of participants’ subjective experiences is required, which we acknowledge as a limitation of the present study. “

DISCUSSION

22) Interpretation consistent with results, balancing benefits and harms, and considering other relevant evidence

NPT: In addition, take into account the choice of the comparator, lack of or partial blinding, and unequal expertise of care providers or centers in each group

22-i) Restate study questions and summarize the answers suggested by the data, starting with primary outcomes and process outcomes (use)

Restate study questions and summarize the answers suggested by the data, starting with primary outcomes and process outcomes (use).

	1	2	3	4	5	
subitem not at all important	○	○	○	○	○	essential

Does your paper address subitem 22-i? *

Copy and paste relevant sections from the manuscript (include quotes in quotation marks "like this" to indicate direct quotes from your manuscript), or elaborate on this item by providing additional information not in the ms, or briefly explain why the item is not applicable/relevant for your study

"This study investigated adherence and app engagement during and 6 months after an mHealth intervention for people post-stroke or TIA. Adherence to supervised sessions remained high (>75%) across the intervention and more females adhered to the supervised exercise than males. Engagement with self-managed mHealth support was initially high but decreased post-intervention. High adherence during the intervention was associated with maintained physical activity between baseline and the 6-month follow-up, while high post-intervention engagement was associated with maintained physical activity between the 6- and 12-month follow-ups."

22-ii) Highlight unanswered new questions, suggest future research

Highlight unanswered new questions, suggest future research.

	1	2	3	4	5	
subitem not at all important	○	○	○	○	○	essential

Does your paper address subitem 22-ii?

Copy and paste relevant sections from the manuscript (include quotes in quotation marks "like this" to indicate direct quotes from your manuscript), or elaborate on this item by providing additional information not in the ms, or briefly explain why the item is not applicable/relevant for your study

"Several aspects of the self-managed mHealth support could be improved to enhance the app engagement in the current mHealth intervention, as well as long-term engagement in physical activity beyond the intervention period. Personalized app content commonly benefits app engagement [41]. However, despite access to an activity diary with self-selected activities and individually prescribed exercises, only 28% (12/43) and 10% (5/50), respectively, frequently engaged with these modules in the present study. Engaging mHealth features, such as prompts, gamification, and regular content updates, have been shown to significantly enhance app engagement [19, 41, 42]. These elements were not incorporated into the current intervention; however, integrating them in future development could be a valuable strategy to encourage maintained app engagement among individuals post-stroke or TIA. Another promising approach to enhance engagement in people post-stroke or TIA, particularly those with lower digital health literacy, is to develop strategies that actively involve caregivers in participating in the mHealth intervention and mediating treatment delivery. This approach has been tested in on-site interventions [45] but has been rarely implemented in digital formats in stroke trials. Furthermore, a potential refinement of the intervention design could be development of distinct phases, each with specific goals for supporting behavior change. This might include an initiation phase focusing on establishing routines for exercise and physical activity (like the present intervention), followed by a maintenance phase in which therapist support occurs less frequently. This design could be especially important for people post-stroke or TIA, who often require long-term support to establish and maintain behavior change [39]. Advancing toward more independent self-management of physical activity would also require extending the intervention's focus to support participants in developing strategies for sustaining behavior change after the intervention, which should be embedded within the individual counseling and educational materials. We believe that refinement of the mHealth version of the i-REBOUND intervention should be co-created with people post-stroke or TIA and target multiple domains, including technology, intervention design, and the establishment of clear expectations for adherence and engagement [46]. "

"User logs yielded an in-depth examination of the participants´ mHealth engagement behavior but could not sufficiently capture the multifaceted nature of engagement. For a more comprehensive understanding of engagement, exploration of participants' subjective experiences is required, which we acknowledge as a limitation of the present study. A strength was the 48-week study period, which allowed for a thorough investigation of changes in adherence and engagement patterns over time. However, adherence and engagement levels >80% were considered 'high', for more substantiated levels, future studies should determine adequate levels for effective mHealth engagement and adherence and set a predefined benchmark [43]."

20) Trial limitations, addressing sources of potential bias, imprecision, and, if relevant, multiplicity of analyses

20-i) Typical limitations in ehealth trials

Typical limitations in ehealth trials: Participants in ehealth trials are rarely blinded. Ehealth trials often look at a multiplicity of outcomes, increasing risk for a Type I error. Discuss biases due to non-use of the intervention/usability issues, biases through informed consent procedures, unexpected events.

	1	2	3	4	5	
subitem not at all important	○	○	○	○	○	essential

Does your paper address subitem 20-i? *

Copy and paste relevant sections from the manuscript (include quotes in quotation marks "like this" to indicate direct quotes from your manuscript), or elaborate on this item by providing additional information not in the ms, or briefly explain why the item is not applicable/relevant for your study

"The findings should be interpreted cautiously given the relatively small sample size. In addition, the participants were mostly female, highly educated, and with mild stroke symptoms, which limits the generalizability of the findings to other stroke or TIA populations [47]. Furthermore, the 7,000 steps/day cut-off for physical activity, while clinically relevant for optimal cardiovascular risk reduction [35], is a crude measure for detecting change over time. Moreover, participants in the sample exhibited a notably high level of physical activity at baseline, exceeding the average daily step count of 4,078 for individuals with stroke [6]. User logs yielded an in-depth examination of the participants´ mHealth engagement behavior but could not sufficiently capture the multifaceted nature of engagement. For a more comprehensive understanding of engagement, exploration of participants' subjective experiences is required, which we acknowledge as a limitation of the present study. A strength was the 48-week study period, which allowed for a thorough investigation of changes in adherence and engagement patterns over time. However, adherence and engagement levels >80% were considered 'high', for more substantiated levels, future studies should determine adequate levels for effective mHealth engagement and adherence and set a predefined benchmark [43]. "

21) Generalisability (external validity, applicability) of the trial findings

NPT: External validity of the trial findings according to the intervention, comparators, patients, and care providers or centers involved in the trial

21-i) Generalizability to other populations

Generalizability to other populations: In particular, discuss generalizability to a general Internet population, outside of a RCT setting, and general patient population, including applicability of the study results for other organizations

	1	2	3	4	5	
subitem not at all important	○	○	○	○	○	essential

Does your paper address subitem 21-i?

Copy and paste relevant sections from the manuscript (include quotes in quotation marks "like this" to indicate direct quotes from your manuscript), or elaborate on this item by providing additional information not in the ms, or briefly explain why the item is not applicable/relevant for your study

"The findings should be interpreted cautiously given the relatively small sample size. In addition, the participants were mostly female, highly educated, and with mild stroke symptoms, which limits the generalizability of the findings to other stroke or TIA populations [47]."

21-ii) Discuss if there were elements in the RCT that would be different in a routine application setting

Discuss if there were elements in the RCT that would be different in a routine application setting (e.g., prompts/reminders, more human involvement, training sessions or other co-interventions) and what impact the omission of these elements could have on use, adoption, or outcomes if the intervention is applied outside of a RCT setting.

	1	2	3	4	5	
subitem not at all important	○	○	○	○	○	essential

Does your paper address subitem 21-ii?

Copy and paste relevant sections from the manuscript (include quotes in quotation marks "like this" to indicate direct quotes from your manuscript), or elaborate on this item by providing additional information not in the ms, or briefly explain why the item is not applicable/relevant for your study

Ditt svar

OTHER INFORMATION

23) Registration number and name of trial registry

Does your paper address CONSORT subitem 23? *

Copy and paste relevant sections from the manuscript (include quotes in quotation marks "like this" to indicate direct quotes from your manuscript), or elaborate on this item by providing additional information not in the ms, or briefly explain why the item is not applicable/relevant for your study

ClinicalTrials: NCT0511195

24) Where the full trial protocol can be accessed, if available

Does your paper address CONSORT subitem 24? *

Cite a Multimedia Appendix, other reference, or copy and paste relevant sections from the manuscript (include quotes in quotation marks "like this" to indicate direct quotes from your manuscript), or elaborate on this item by providing additional information not in the ms, or briefly explain why the item is not applicable/relevant for your study

Reference: Thurston C, Bezuidenhout L, Humphries S, Johansson S, von Koch L, Häger CK, Holmlund L, Sundberg CJ, Garcia-Ptacek S, Kwak L, Nilsson M, English C, Conradsson DM. Mobile health to promote physical activity in people post stroke or transient ischemic attack - study protocol for a feasibility randomised controlled trial. BMC Neurol. 2023 Mar 28;23(1):124. doi: 10.1186/s12883-023-03163-0. PMID: 36978045; PMCID: PMC10043533.

25) Sources of funding and other support (such as supply of drugs), role of funders

Does your paper address CONSORT subitem 25? *

Copy and paste relevant sections from the manuscript (include quotes in quotation marks "like this" to indicate direct quotes from your manuscript), or elaborate on this item by providing additional information not in the ms, or briefly explain why the item is not applicable/relevant for your study

"This work was supported by grants from the Swedish Research Council (2022-01403), Swedish Research Council for Health, Working Life and Welfare (2021-01018), Center for Innovative Medicine, Region Stockholm (FoUI-960631), Sweden's Innovation Agency (2021-01726), Swedish Stroke Association, Strategic Research Area Health Care Sciences at Karolinska Institutet, and Research School of Health Science at Karolinska Institutet."

The funders had no role in the design, conduct, analysis, or interpretation of the result of this study.

X27) Conflicts of Interest (not a CONSORT item)

X27-i) State the relation of the study team towards the system being evaluated

In addition to the usual declaration of interests (financial or otherwise), also state the relation of the study team towards the system being evaluated, i.e., state if the authors/evaluators are distinct from or identical with the developers/sponsors of the intervention.

	1	2	3	4	5	
subitem not at all important	○	○	○	○	○	essential

Does your paper address subitem X27-i?

Copy and paste relevant sections from the manuscript (include quotes in quotation marks "like this" to indicate direct quotes from your manuscript), or elaborate on this item by providing additional information not in the ms, or briefly explain why the item is not applicable/relevant for your study

The STAAR app is managed by the MedTech company Empowerment Health. Empowerment Health had no involvement in the design, conduct, analysis, or interpretation of the results of this study.

About the CONSORT EHEALTH checklist

As a result of using this checklist, did you make changes in your manuscript? *

- ○ yes, major changes
- ◉ yes, minor changes
- ○ no

What were the most important changes you made as a result of using this checklist?

The most important changes included a more comprehensive description of the consent procedures (item 4a-iii), the company that managed the mHealth app (item 5-v), the intervention components and the STAAR application interface, as well as a clarification of the intended dose of adherence and engagement.

How much time did you spend on going through the checklist INCLUDING making changes in your manuscript *

We spent 15 hours thoroughly going through the checklist.

As a result of using this checklist, do you think your manuscript has improved? *

- (x) yes
- ( ) no
- ( ) Övrigt:

Would you like to become involved in the CONSORT EHEALTH group?

This would involve for example becoming involved in participating in a workshop and writing an "Explanation and Elaboration" document

- ( ) yes
- (x) no
- ( ) Övrigt:

Rensa markering

Any other comments or questions on CONSORT EHEALTH

The submitted manuscript is part of a process evaluation, and this specific study aims to explore adherence to and engagement with an mHealth intervention. Since this study was not an efficacy trial, certain items in the CONSORT-EHEALTH checklist were not applicable, and some aspects are reported elsewhere, such as in the study protocol or related studies. However, several items were highly relevant, and thanks to the CONSORT-EHEALTH checklist, we identified content in the manuscript that needed clarification and improvement.

STOP - Save this form as PDF before you click submit

To generate a record that you filled in this form, we recommend to generate a PDF of this page (on a Mac, simply select "print" and then select "print as PDF") before you submit it.

When you submit your (revised) paper to JMIR, please upload the PDF as supplementary file.

Don't worry if some text in the textboxes is cut off, as we still have the complete information in our database. Thank you!

Final step: Click submit !

Click submit so we have your answers in our database!

Skicka

Rensa formuläret

Skicka aldrig lösenord med Google Formulär

Det här innehållet har varken skapats eller godkänts av Google. - Användarvillkor - Integritetspolicy

Ser det här formuläret misstänkt ut? Rapport

Google Formulär
